# Effect of *n*-Octadecylphosphonic Acid Coating on Zeolite 5A for Adsorptive Separation of Carbon Dioxide and Propylene

**DOI:** 10.3390/molecules31030564

**Published:** 2026-02-05

**Authors:** Magdy Abdelghany Elsayed, Shixue Zhou, Chengdong Zhang, Kun Zhang

**Affiliations:** 1 College of Energy and Mining Engineering, Shandong University of Science and Technology, Qingdao 266590, China; magdyabdelghany10@163.com (M.A.E.); zhangchengdong11@126.com (C.Z.); kunzhang87@163.com (K.Z.); 2 Department of Mining and Petroleum Engineering, Faculty of Engineering, Al-Azhar University, Cairo 11884, Egypt; 3 College of Chemical and Biological Engineering, Shandong University of Science and Technology, Qingdao 266590, China

**Keywords:** zeolite 5A, surface modification, adsorptive separation, ideal selectivity, diffusion analysis

## Abstract

Understanding the competitive adsorption mechanism is crucial for the rational design of CO_2_ adsorbents. In this work, the surface of zeolite 5A was modified with varying concentrations of *n*-octadecylphosphonic acid (ODPA) to enhance the adsorptive separation of CO_2_ over C_3_H_6_. With a 0.01 mol/L concentration of ODPA, the modified zeolite 5A achieves CO_2_/C_3_H_6_ ideal selectivity over 73 at 298 K, a substantial improvement over the pristine zeolite 5A, which exhibits a selectivity of 6.07. The Sips isotherm model provides an excellent fit to the experimental data, offering insights into the adsorption mechanism, with a calculated enthalpy change of −30.70 kJ/mol for CO_2_ and −16.54 kJ/mol for C_3_H_6_, along with favorable Gibbs free energy changes ranging from −9.00 to −3.54 kJ/mol for CO_2_ and −4.96 to −2.04 kJ/mol for C_3_H_6_ over the temperature range of 298–373 K. Kinetic analysis reveals faster diffusion in pristine zeolite 5A; however, surface modification significantly enhances CO_2_/C_3_H_6_ selectivity while maintaining balanced adsorption capacity. Adsorption uptakes of CO_2_ and C_3_H_6_ in pristine zeolite 5A follow the pseudo-first-order model and pseudo-second-order model, respectively. Pristine zeolite 5A shows rapid adsorption, with a CO_2_ adsorption capacity of 4.10 mmol/g with a rate constant of 2.60 min^−1^, and a C_3_H_6_ adsorption capacity of 1.99 mmol/g with a rate constant of 0.34 min^−1^. The modification with ODPA increases adsorption energy barriers, with CO_2_ activation energy reaching 5.18 kJ/mol and C_3_H_6_ activation energy up to 15.63 kJ/mol, while tetrahydrofuran washing restores site accessibility, demonstrating tunable diffusion and adsorption behavior. These findings lay the foundation for designing high-efficiency, and selective adsorbents through targeted surface engineering.

## 1. Introduction

Continuous increase of carbon dioxide (CO_2_) levels in the atmosphere, primarily driven by anthropogenic activities such as fossil fuel consumption, cement production, and land-use changes, has intensified global efforts to develop efficient adsorbents for application in CO_2_ capture technologies to mitigate the significant implications for climate change [1,2]. These global efforts resulted in a diverse array of materials, including activated carbons [3,4], lithium ceramics [5,6], ionic liquids [7,8], amines [9,10,11], molten salts [12,13], polymeric membranes [14,15], hydrocarbon surfactants [16,17], and zeolites. Among these, zeolites have been extensively investigated for their potential to selectively and efficiently capture CO_2_ [18,19]. Currently, CO_2_ separation is primarily achieved through four key techniques—absorption, adsorption, cryogenic separation, and membrane separation [20]. Choice of a specific method depends on a variety of factors, such as the emission source and scale, the required CO_2_ purity, and the intended utilization pathway [21]. Among these, absorption and adsorption have emerged as the most widely adopted techniques for CO_2_ separation [22]. Absorption involves dissolving CO_2_ into a solvent, relying either on physical retention or chemical reactions depending on solvent nature. In contrast, adsorption depends on interactions between CO_2_ molecules and the surface of solid adsorbents via physisorption or chemisorption, influenced by surface characteristics of the adsorbents [23,24]. Three primary CO_2_ capture routes in which adsorption dominates the field: post-combustion, pre-combustion, and oxy-fuel combustion [25,26]. Post-combustion capture is particularly attractive because it allows existing infrastructure to be retrofitted without major changes to combustion systems, making it highly relevant for industrial applications. A variety of porous solid adsorbents including zeolites, metal–organic frameworks (MOFs), activated carbon, graphene, silica, transition metal oxides, alumina, and organic polymers, have demonstrated effective CO_2_ capture performance under post-combustion conditions [27]. Zeolites stand out due to their well-ordered microporous architecture, high surface area, and ion-exchange capability, all of which enable highly selective CO_2_ adsorption [28]. Zeolites are broadly classified into natural and synthetic types, with their performance closely tied to their crystal structure and chemical composition [29]. The CO_2_ adsorption capacity of zeolites is governed by several parameters, including surface area, crystal topology, and both the nature and accessibility of exchangeable cations. However, adsorption performance can deteriorate due to the competitive adsorption of gases such as CH_4_, N_2_, and H_2_O, as well as under elevated temperatures and reduced pressures [30,31,32]. Specific zeolite frameworks such as 13X [33], Na-Y [34], ZSM-5 [35], and SSZ-13 [36] have been tailored through cation modification and porosity enhancement to improve their CO_2_ capacity. Notably, higher aluminum content generally strengthens the electrostatic interactions between CO_2_ molecules and the zeolite framework. To further enhance CO_2_ selectivity and capacity, various physical and chemical modification techniques have been applied to zeolites [37]. Surface functionalization with amines has successfully improved adsorption in zeolites like HEU, FAU, BEA, and LTA [38,39,40,41,42]. Ion exchange with alkali, alkaline earth, or transition metal cations has been used to tune the electrostatic environment within pores, especially in FAU, CHA, KFI, and BEA frameworks [38,43,44,45]. Acid treatments adjust both acidity and pore dimensions in FAU zeolites [33,46], while surfactant-assisted methods optimize surface morphology in clinoptilolite [47]. Silica-based modifications, including composite silica-zeolite systems, enhance structure stability and adsorption performance in zeolite types like KFI, BEA, LTA, and MFI [35,48]. Advanced composite strategies, such as carbon-zeolite hybrids and MOF-zeolite frameworks, combine the strengths of multiple adsorbent classes to improve performance under humid and variable conditions [49,50,51]. Additionally, nanosizing approaches boost surface area and mass transfer rates in zeolites such as LTL and LTN [39,52]. Recent studies indicate the potential of zeolites modification to significantly improve adsorption performance. For instance, Sun et al. [53] demonstrated that ion-exchange for SSZ-13 zeolite with transition metal cations (e.g., Co^2+^ and Ni^2+^) exhibited superior CO_2_ capacity (4.49 and 4.45 mmol/g, respectively) and high CO_2_/N_2_ selectivity at 273 K and 100 kPa. Chudasama et al. [54] enhanced O_2_/Ar and O_2_/N_2_ selectivity by coating zeolite 4A with silica from tetraethyl orthosilicate. Xu et al. [55] reported a doubling of CO_2_/N_2_ selectivity in β-zeolite through functionalization with monoethanolamine. Ellis et al. [56] showed that phosphonic acid modifications on zeolite 5A effectively tune gas diffusion properties while preserving structure integrity, as supported by XRD, BET, and STEM-EDS analysis.

In this context, zeolite 5A represents a compelling candidate for further functionalization studies, given its well-defined pore structure and performance in gas separation. Still, there is a need to systematically investigate the influence of surface modification strategies particularly with organic phosphonic acid derivatives such as *n*-octadecylphosphonic acid (ODPA) on CO_2_ and hydrocarbon (C_3_H_6_) adsorption, with the goal of optimizing selective gas separation for industrial applications. ODPA is selected as a surface modifier because phosphonic acids are known to chemisorb strongly onto metal-oxide-containing surfaces, forming robust monolayers that allow the fine-tuning of surface polarity and framework-adsorbate interactions. The introduction of ODPA is therefore expected to modulate both time-dependent uptake and thermodynamics, enhancing the discrimination between molecules of similar sizes but differing quadrupole moments or polarizability [57,58]. This strategy is supported by prior reports in which phosphonic-acid functionalization improved separation performance in hydrocarbon adsorption systems, including enhanced selectivity due to controllable interfacial chemistry [59,60,61]. The markedly different adsorption affinities of CO_2_ and C_3_H_6_ towards porous solids provide an opportunity to design adsorbents capable of delivering high-purity hydrocarbon products while simultaneously enabling effective CO_2_ capture. Previous studies have demonstrated the feasibility of such separations using advanced adsorbents, including metal–organic frameworks and polydopamine-derived carbons, which exhibit strong CO_2_ binding but minimal capacity of light hydrocarbons, thereby affording high selectivity and excellent cycling stability under practical operating conditions [62,63,64].

This work focuses on the adsorptive separation of CO_2_ from C_3_H_6_ (propylene) owing to the critical importance of efficient CO_2_ capture from hydrocarbon-containing process streams. Such separation is central for mitigating greenhouse gas emissions, improving energy efficiency, and enabling downstream gas purification in carbon-capture and storage (CCS) schemes. Furthermore, C_3_H_6_ is an essential petrochemical product, and its selective recovery from CO_2_-rich mixtures is highly relevant for process intensification and product purification. Therefore, we investigated the adsorption performance of CO_2_ and C_3_H_6_ in pristine and ODPA-modified zeolite 5A under controlled operation conditions. The objectives are (i) comprehensive physicochemical characterization of pristine and modified zeolite 5A, (ii) evaluation of adsorption isotherms at different distinct temperatures, (iii) application of established isotherm models to elucidate adsorption mechanisms, (iv) determination of adsorption enthalpies to gain thermodynamic insights, and (v) analysis of adsorption uptake using intraparticle diffusion and kinetic models to estimate rate constant and activation energy. Additionally, the impact of ODPA coverage on selective CO_2_ adsorption over C_3_H_6_ is assessed, providing critical insight into the design of functionalized zeolite adsorbents for greenhouse gas mitigation. These findings aim to enhance the understanding of structure–property relationships in surface-modified zeolites and contribute to the advancement of efficient CO_2_ capture materials with potential for industrial application.

## 2. Results and Discussion

### 2.1. Characterization of Adsorbent

#### 2.1.1. XRD Analysis

**Figure 1:**
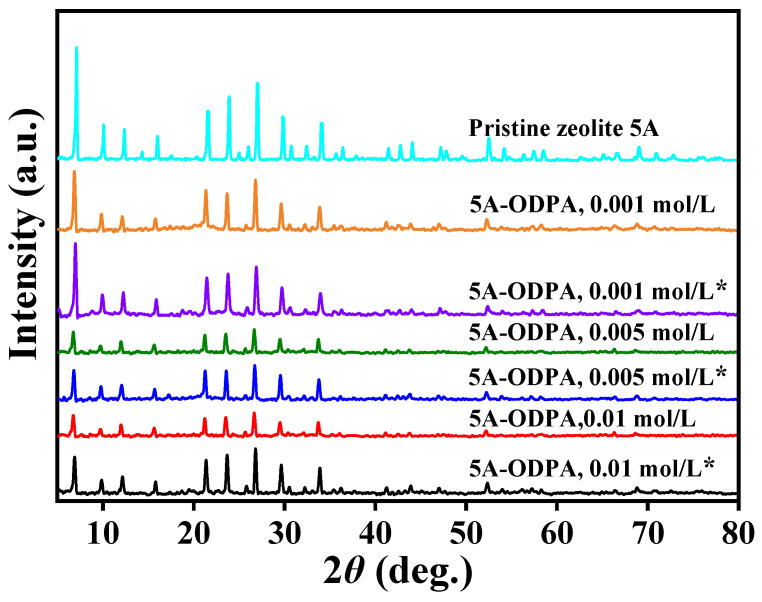
XRD patterns of pristine and modified zeolite 5A; materials washed with THF after modification are marked with an asterisk (*).

X-ray diffraction (XRD) patterns of pristine and the ODPA-modified zeolite 5A materials under different concentrations are shown in Figure 1. The topmost pattern corresponds to pristine zeolite 5A and serves as the reference for other materials. Remaining patterns represent modified zeolite 5A unwashed and washed with tetrahydrofuran (THF), and the latter are indicated by an asterisk (*). Pristine zeolite 5A exhibits sharp peaks indexed to planes such as (100), (110), (111), (210), (221), (331), (320), (321), (410), (411), (420), (332), and (550), confirming high crystallinity. Across all modified materials, the main diffraction peaks remain consistent to the pristine zeolite 5A, indicating that the crystalline framework is preserved after modification. Minor variations in peak intensity occur, likely due to ODPA coverage on zeolite surface. THF-washed materials show more distinct intensity, suggesting effective removal of excess ODPA, without compromising the structure integrity of the modified zeolite.

#### 2.1.2. FTIR Spectrum

**Figure 2:**
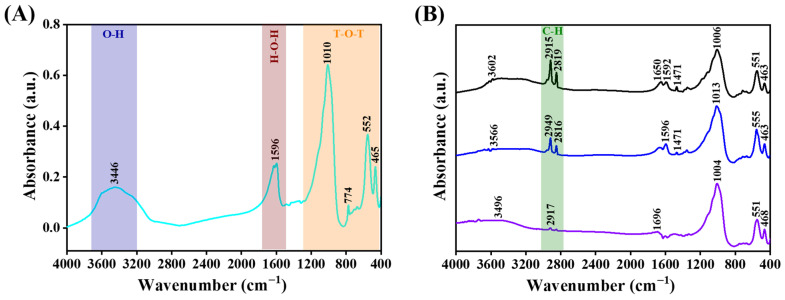
FTIR spectra of pristine and modified zeolite 5A: (**A**) pristine zeolite 5A and (**B**) modified zeolite 5A with varying ODPA concentrations after THF washing; black line represents 0.01 mol/L ODPA-modified zeolite 5A, blue line shows 0.005 mol/L ODPA-modified zeolite 5A, and purple line represents 0.001 mol/L ODPA-modified zeolite 5A. T-O-T, the essential oxygen-bridged linkage defining the zeolite structure; T represents framework atoms, specifically Si or Al.

FTIR spectra for pristine and the ODPA-modified zeolite 5A materials at different ODPA concentrations are presented in Figure 2 and Figure S1. The comparison of pristine zeolite 5A with the modified materials after THF washing is shown in Figure 2, indicating changes in the surface bonding environment and confirming the removal of physiosorbed ODPA. In contrast, Figure S1 shows the spectrum of the modified materials, reflecting the initial state of ODPA incorporation before THF washing.

FTIR spectra of pristine and the ODPA-modified zeolite 5A materials (Figure 2) confirm the preservation of the aluminosilicate framework and successful surface functionalization. The characteristic bands of pristine zeolite 5A (Figure 2A), such as the T-O bending vibration at 463 cm^−1^ (T = Si or Al) [65], the double 4-ring (D4R) vibration at 555 cm^−1^ [66], and the Si-O-Si/Si-O-Al stretching modes between 668 and 774 cm^−1^ [67], demonstrate the structure integrity of the framework. The FTIR spectrum for pristine zeolite 5A also shows a broad band at approximately 3450 cm^−1^, which is attributed to O-H stretching vibration from both surface hydroxy groups and adsorbed water molecules. Furthermore, the presence of molecular water is confirmed by a distinct band at 1596 cm^−1^, corresponding to the H-O-H bending mode [68,69]. This indicates that the material surface contains both inherent hydroxy groups and physiosorbed water. The high absorbance at 1596 cm^−1^ indicates a significant amount of molecular water on the surface, which may hinder the covalent grafting of ODPA if not controlled. Therefore, pre-drying and anhydrous solvents are employed to ensure efficient formation of Al-O-P linkage.

After ODPA modification (Figure 2B and Figure S1), the emergence of C-H stretching at 2915 and 2849 cm^−1^, along with CH_2_/CH_3_ bending modes at 1471 cm^−1^ and 1369 cm^−1^, provides clear evidence of alkyl chain attachment [70,71]. An increase in O-H stretching absorbance at 3450 cm^−1^ is observed upon ODPA modification, indicating the attachment of ODPA on zeolite surface. At low ODPA concentrations (0.001 mol/L), the reversibility of this peak upon washing (Figure 2B) suggests ODPA physisorption, whereas their persistence at higher concentrations (0.01 mol/L) confirms ODPA chemisorption.

Notably, comparing pristine and the ODPA-modified zeolite 5A materials washed with THF (Figure 2), the absorbance at 1596 cm^−1^ is higher in the pristine material due to fully accessible molecular water. After washing, partial exposure of the surface sites leads to the reappearance of this band in the ODPA-modified zeolite 5A materials, but lower absorbance is consistent with the majority of surface sites remaining covered by the grafted ODPA. Furthermore, the linkages between ODPA and zeolite 5A surface are inferred from the persistence of ODPA characteristic peaks after THF washing. Overall, these observations confirm that the ODPA layer is chemically stable on the zeolite surface.

#### 2.1.3. Thermal Analysis

**Figure 3:**
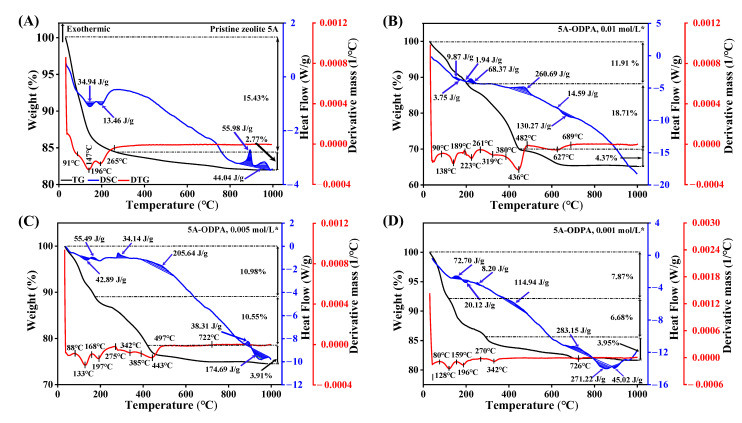
TG, DTG, and DSC curves of pristine and modified zeolite 5A: (**A**) pristine zeolite 5A, (**B**) 0.01 mol/L ODPA-modified zeolite 5A, (**C**) 0.005 mol/L ODPA-modified zeolite 5A, and (**D**) 0.001 mol/L ODPA-modified zeolite 5A. Asterisk (*) denotes the THF-washed materials after modification.

TG curves provide the overall mass-loss profile, enabling quantification of water, organics, and residual species at each stage. DTG curves, which are the derivative of the TG data, indicate the exact temperatures at which decomposition and transition events occur. DSC profiles indicate the enthalpy changes associated with thermal events, distinguishing endothermic and exothermic processes. Figure 3 shows that ODPA modification on zeolite 5A reduces water adsorption and shifts water loss temperatures, indicating that ODPA coating alters the pore accessibility for water and minimizes water-zeolite interactions [72].

TGA curve of pristine zeolite 5A (Figure 3A) shows a total weight loss of 18.20%, occurring in two regions. The low temperature region accounts for desorption of physiosorbed water, and the high temperature region is attributed to dihydroxylation and the release of structurally bound water [73]. The DSC curve indicates an endothermic peak at 150 °C with an enthalpy change of 34.94 J/g corresponding to physisorbed water removal. A second endothermic peak at 200 °C with an enthalpy of 13.46 J/g corresponds to the release of structure water. Exothermic peaks are observed at 900 °C (−55.98 J/g) and 950 °C (−44.04 J/g), indicating the change in the crystalline structure of the zeolite. These results reflect sequential processes of water removal and crystal phase change.

For the 0.01 mol/L ODPA unwashed material as shown in Figure S2A, three distinct weight-loss regions are observed in the TGA curve: removal of physisorbed water below 200 °C, desorption of physisorbed and weakly bound ODPA up to 500 °C, and degradation of residual organics above 500 °C. Corresponding DSC signals indicate dehydration (~140 °C), ODPA-related transition (~500 °C), and high-temperature framework changes near 900–950 °C. The TGA curve of 0.01 mol/L ODPA-modified zeolite 5A material after THF washing shows more gradual (Figure 3B), with increased mass loss between 200 and 500 °C, which is attributed to strongly bound ODPA, while DSC peaks shift to 215, 450, and 650 °C, indicating different ODPA transition behavior.

A similar trend is observed for the 0.005 and 0.001 mol/L ODPA-modified zeolite 5A materials. Prior to washing (Figure S2B,C), both materials display the characteristic three-step mass-loss sequence corresponding to dehydration, ODPA transition, and framework change, accompanied by multiple DSC signals associated with ODPA transition (450–550 °C) and framework change (900–950 °C). After THF washing (Figure 3C,D), low-temperature mass loss is reduced, and the ODPA-related region becomes narrower, indicating efficient removal of weakly physisorbed species while retaining strongly bound ODPA. The washed materials exhibit streamlined DSC signatures with attenuated transition peaks and more coherent high-temperature endotherms, reflecting stabilized thermal behavior.

The behaviors from the high ODPA concentration are quite different from the low concentration. From 0.01 mol/L ODPA, the high initial ODPA coverage leads to a large amount of strongly bound ODPA remaining after washing, resulting in increased mass loss in the 200–500 °C range. In contrast, from low ODPA concentration, the material retains less ODPA after washing, which accounts for the reduced and narrowed ODPA-related mass-loss regions and attenuated DSC peaks. Collectively, the TGA-DSC results demonstrate that ODPA remains strongly anchored to the zeolite after washing (Table S1).

#### 2.1.4. XPS Analysis

**Figure 4:**
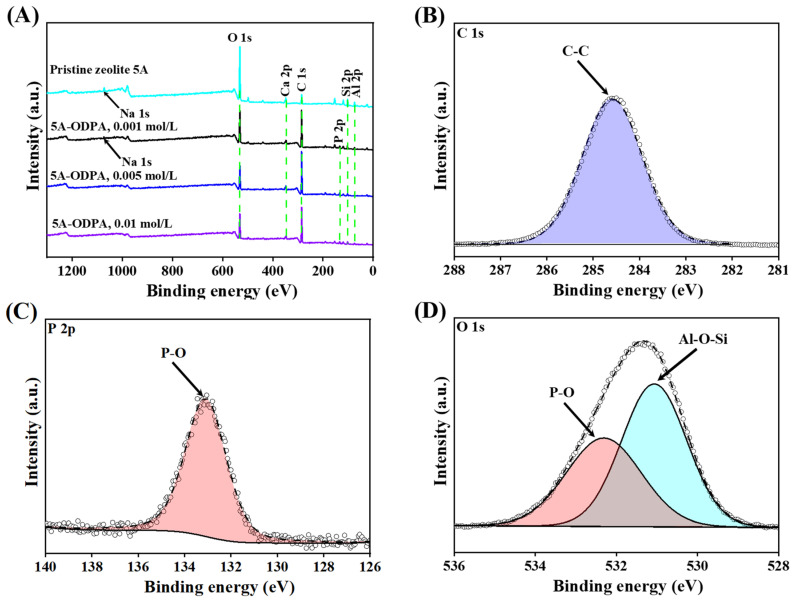
XPS spectra of pristine and modified zeolite 5A without THF washing: (**A**) full-range scan before and after modification, (**B**) high-resolution C 1s, (**C**) P 2p, and (**D**) O 1s spectra of modified zeolite 5A with 0.01 mol/L ODPA.

XPS spectra of pristine and the ODPA-modified zeolite 5A materials are shown in Figure 4A–D. In the full-range spectra (Figure 4A), the pristine zeolite 5A shows characteristic peaks of sodium (Na 1s), oxygen (O 1s), calcium (Ca 2p), aluminum (Al 2p), and silicon (Si 2p), along with a small carbon peak (C 1s) likely from surface contamination [74]. After surface modification with ODPA (0.01, 0.005, and 0.001 mol/L), a new peak at ∼133 eV from P 2p appears. The carbon peak (C 1s) grows stronger, reflecting increased organic material on the surface. Simultaneously, the zeolite framework peaks (O 1s, Ca 2p, Al 2p, Si 2p) decrease, suggesting that ODPA is increasingly masking the zeolite structure, especially at higher concentrations. Furthermore, the atomic composition of P (from ODPA) and C (from the aliphatic chain of OPDA) increase as a function of OPDA concentration (Table S2), indicating the increase in ODPA on the zeolite surface.

The C 1s spectrum of the modified zeolite 5A from a 0.01 mol/L ODPA concentration reveals a prominent peak at 284.60 eV corresponding to C-C bonding in the ODPA molecules (Figure 4B) [75]. The P 2p spectrum (Figure 4C) reveals a prominent peak at 133.08 eV, which corresponds to P-O groups, typically associated with phosphorus bonded to the zeolite framework through P-O-Al linkage, confirming the successful surface coating of the phosphonic acid [76]. Furthermore, the O 1s spectrum (Figure 4D) exhibits two principal peak components at 531.07 eV and 532.34 eV, corresponding to Al-O-Si and P-O species in the ODPA-coated zeolite, respectively, similar to findings from Yulin et al. [77]. As a result, the modification involved the reaction between the phosphonic acid group of ODPA and surface hydroxyl groups of the zeolite 5A, forming stable P-O-Al bonds. This detailed XPS analysis elucidates the chemical environment of carbon, phosphorus, and oxygen within the modified zeolite matrix, providing insights into the surface chemistry of ODPA functionalized zeolite surface.

#### 2.1.5. SEM-EDS Examination of Materials

**Figure 5:**
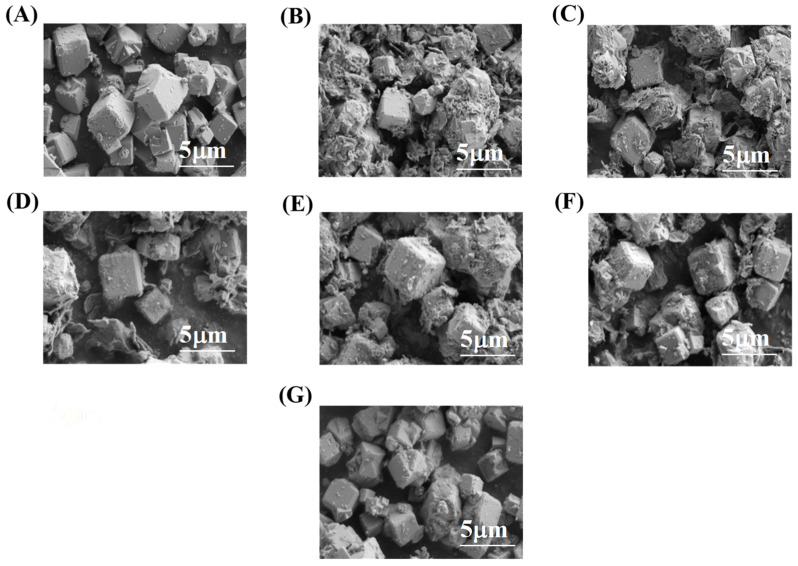
SEM images of pristine and modified zeolite 5A: (**A**) pristine zeolite 5A, (**B**) 0.01 mol/L ODPA-modified zeolite 5A (unwashed), (**C**) 0.01 mol/L ODPA-modified zeolite 5A (washed), (**D**) 0.005 mol/L ODPA-modified zeolite 5A (unwashed), (**E**) 0.005 mol/L ODPA-modified zeolite 5A, (**F**) 0.01 mol/L ODPA-modified zeolite 5A (unwashed), and (**G**) 0.001 mol/L ODPA-modified zeolite 5A).

The SEM images (Figure 5) show the effect of ODPA modification with different concentrations and subsequent washing with THF on the zeolite surface morphology. Pristine zeolite 5A is shown in Figure 5A, characterized by well-defined cubic crystals with smooth surfaces and sharp edges, representing the basic morphology of the pristine zeolite material, while Figure 5B depicts 0.01 mol/L modified zeolite 5A without THF washing, where significant morphological changes are observed, including heavily coated crystals and particle agglomeration, indicating a thick ODPA layer masking the sharp edges. Moreover, Figure 5C shows the effect of THF washing on 0.01 mol/L modified zeolite 5A, revealing partial removal of the ODPA layer, which restores some of the original cubic features; however, the residue suggests that a portion of the ODPA remains chemically adsorbed onto the zeolite 5A surface, indicating strong interactions that resist removal even with solvent washing. Figure 5D indicates 0.005 mol/L modified zeolite 5A without washing, exhibiting a thinner ODPA layer compared to Figure 5B, with the original crystal structure more discernible, yet some roughness persists. After THF washing, Figure 5E shows clear crystal outlines, indicating further reduction in the ODPA layer. Figure 5F and Figure 5G represent the effect of 0.001 mol/L modification without and with THF washing, respectively. Figure 5F demonstrates minimal coating and preservation of crystal morphology, while Figure 5G reveals the low coverage of ODPA on the zeolite surface, nearly restoring pristine morphology. This indicates that too low of a concentration of ODPA is not sufficient for zeolite surface modification.

The EDS analysis is performed to evaluate changes in elemental composition following ODPA modification and subsequent THF washing, with the quantitative elemental data extracted from the spectra in Figures S3 and S4 and summarized in Table 1. The elemental mapping images (Figures S5 and S6) confirm uniform spatial distribution of both the framework elements and the ODPA-derived species across the particle surfaces. The pristine zeolite 5A displays the expected framework composition, dominated by O, Al, Si, Na, and Ca. Upon ODPA treatment, the unwashed materials exhibit marked increases in C and P contents, confirming successful ODPA deposition on the external surface. The magnitude of this increase scales with ODPA concentration, with the 0.01 mol/L material showing the highest C (32.09 wt%) and P (2.70 wt%) coverage. After THF washing, all materials show a clear and systematic reduction in both elements, consistent with efficient removal of physically adsorbed ODPA. The proportional decrease in C and P across concentrations confirms that both elements originate from ODPA, and that washing selectively removes the loosely bound fraction.

Slight increases in the relative abundance of these elements after washing are attributed to the re-exposure of the underlying aluminosilicate surface as physisorbed ODPA is removed. Importantly, the Si/Al molar ratio remains effectively unchanged across all materials, demonstrating that the zeolite framework remains intact and unaltered, with cation distribution within the zeolite remaining stable under all studied conditions. ODPA adsorption increases the C and P content proportionally to precursor concentration, whereas THF washing selectively removes the physically adsorbed fraction while preserving the chemically anchored ODPA species. The composition analysis confirms that the ODPA modification process does not perturb the bulk structure of zeolite 5A and that the washing step effectively differentiates between strongly and weakly bound ODPA.

#### 2.1.6. Solid-State NMR Analysis

**Figure 6:**
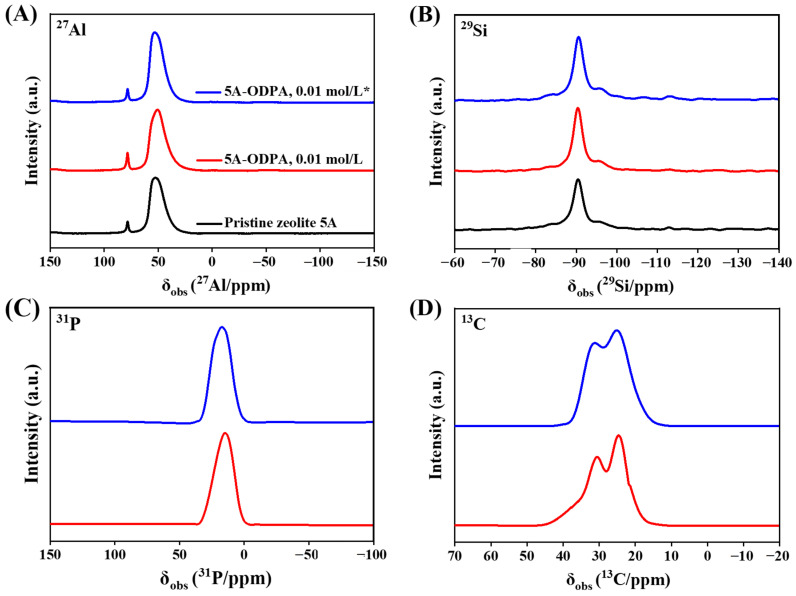
Solid-state NMR spectra of pristine and modified zeolite 5A samples: (**A**) ^27^Al NMR, (**B**) ^29^Si NMR, (**C**) ^31^P NMR, and (**D**) ^13^C NMR. Spectra are shown for pristine zeolite 5A (black), 0.01 mol/L ODPA-modified zeolite 5A unwashed (red), and 0.01 mol/L ODPA-modified zeolite 5A with THF washed (blue). Asterisk (*) denotes the THF-washed materials after modification.

Solid-state NMR spectra of pristine and 0.01 mol/L ODPA-modified zeolite 5A are shown in Figure 6. The spectra confirm the preservation of the zeolite framework after ODPA modification and successful surface functionalization. The ^27^Al MAS NMR spectra exhibit two characteristic resonances centered at approximately 53 and 78 ppm. The signal at ~53 ppm corresponds to tetrahedrally coordinated framework aluminum, while the resonance at ~78 ppm is assigned to extra-framework four-coordinated Al(OH)_4_^−^ species, consistent with references [78,79,80]. The tetrahedral framework aluminum resonance exhibits a negligible chemical shift change (Δδ < 0.6 ppm), indicating that ODPA modification does not induce framework collapse or significant structural distortion. Quantitative analysis of the ^27^Al MAS NMR spectra shows that pristine zeolite 5A contains predominantly tetrahedral framework aluminum, with only a minor fraction of extra-framework aluminum (4.48:95.52 for extra-framework:tetrahedral). After ODPA modification, the fraction of extra-framework aluminum increases slightly to 5.37:94.63. Subsequent washing reduces the extra-framework aluminum content to 4.12:95.88, while the tetrahedral fraction returns to a value comparable to that of the pristine zeolite 5A material. These results demonstrate that ODPA modification does not cause irreversible dealumination and that the zeolite 5A framework remains intact. The ^29^Si spectra exhibit a singular, sharp resonance at approximately −90.5 ppm, assigned exclusively to the Q^4^(4Al) silicon environment of the intact LTA-type framework, confirming that the modification is a surface-specific process [81]. The ^31^P NMR spectrum provides direct evidence of successful grafting, marked by the appearance of a resonance at 15.10 ppm. This chemical shift is diagnostic of a phosphonate group (R-PO(OH)_2_) chemically bound to the hydroxylated aluminosilicate surface of zeolite 5A [82]. After THF washing, this resonance shifts to 17.33 ppm, indicating the removal of physisorbed ODPA molecules and the formation of a more ordered surface monolayer stabilized by stronger bidentate coordination. The formation of a covalently anchored organic monolayer is further corroborated by ^13^C NMR. The spectrum of the ODPA-modified material before washing exhibits a composite resonance with components at 24.7 and 30.7 ppm, assigned to the α-methylene carbon (P-CH_2_-) and the inner methylene (CH_2_) groups of the octadecyl chain, respectively [83]. Following THF washing, this signal persists with components shifted to 25.3 and 31.5 ppm with reduced overall intensity. The intensity reduction is consistent with the removal of physisorbed molecules, as indicated by the ^31^P NMR shift, while the persistence of the sharp signal confirms that a stable, covalently grafted monolayer remains on the zeolite surface.

The location and state of the ODPA modifier are elucidated through complementary characterization. SEM/EDX analysis confirms the presence of phosphorus-containing species distributed on the zeolite surface. Crucially, solid-state NMR spectroscopy provides definitive molecular-level insight: the pristine ^27^Al and ^29^Si MAS NMR spectra are fully preserved after modification, demonstrating that the internal microporous framework remains intact and is not penetrated by ODPA. Concurrently, the emergence of 31P and ^13^C NMR signals confirms the presence of ODPA, with the ^31^P chemical shift (~15–17 ppm) indicating a covalently bound phosphonate monolayer on the surface.

### 2.2. Adsorption Isotherms

**Figure 7:**
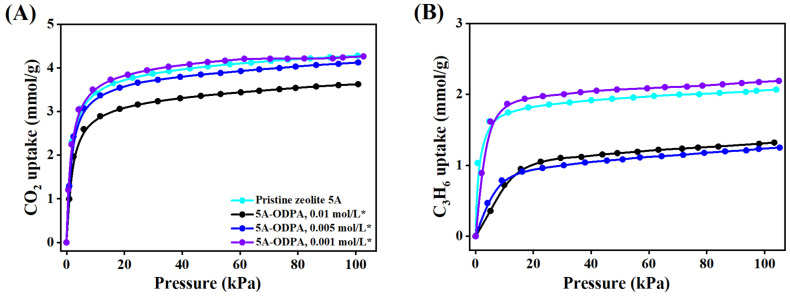
Adsorption isotherms of (**A**) CO_2_ and (**B**) C_3_H_6_ at 298 K for pristine and ODPA-modified zeolite 5A with different ODPA concentrations. Asterisk (*) denotes the THF-washed materials after modification.

Single-component adsorption isotherms of CO_2_ and C_3_H_6_ in pristine and ODPA-modified zeolite 5A materials at different temperatures are shown in Figure 7 and Figures S7–S10. The capacities of both CO_2_ and C_3_H_6_ decrease with increasing temperature due to high molecular kinetic energy, indicating that adsorption is favored at low temperatures where molecules have low kinetic energy and strong interactions with the adsorbent. At 298 K and 100 kPa, as shown in Figure 7A, pristine zeolite 5A exhibits adsorption capacities of 4.28 mmol/g for CO_2_ and 2.07 mmol/g for C_3_H_6_. The modification of 0.001 mol/L ODPA with THF washing results in an almost identical CO_2_ capacity of 4.26 mmol/g and C_3_H_6_ capacity of 2.19 mmol/g (Figure 7B), indicating that such a low ODPA concentration preserves the adsorption performance and is insufficient to produce meaningful surface modification.

As the ODPA concentration increases (0.005 and 0.01 mol/L), the washed materials show gradually decreased adsorption, with CO_2_ capacities of 4.12 and 3.62 mmol/g, and C_3_H_6_ capacities of 1.33 and 1.24 mmol/g, respectively, as shown in Figure 7A,B. Despite this decline, the reduction is more significant for C_3_H_6_ than for CO_2_, suggesting that CO_2_ adsorption remain largely accessible while C_3_H_6_ diffusion is increasingly restricted by surface-bound ODPA. This indicates an enhanced CO_2_/C_3_H_6_ selectivity, particularly in the 0.01 mol/L washed ODPA material, which balances reasonable adsorption capacity with improved selectivity. The superior performance of the washed materials is attributed to the THF washing step, which effectively removes physically adsorbed ODPA and minimizes pore blockage.

In contrast, the unwashed materials consistently show low adsorption capacities, as ODPA occupies pore spaces and partially obstructs diffusion (Figures S7 and S10). At 298 K and 100 kPa, CO_2_ and C_3_H_6_ capacities decrease to 3.77 and 1.15 mmol/g for the 0.005 mol/L unwashed material, and further to 3.00 and 0.98 mmol/g for the 0.01 mol/L unwashed material, as shown in Figure S7A,B. At 373 K, the 0.01 mol/L unwashed material exhibits the lowest adsorption, with CO_2_ and C_3_H_6_ capacities of 1.87 and 0.49 mmol/g, respectively, as shown in Figure S10, indicating the detrimental effect of physically adsorbed ODPA in the absence of washing with THF for modified zeolite 5A. Therefore, the results demonstrate that controlled ODPA functionalization, coupled with THF washing, maintains the adsorption capacity of zeolite 5A and improves CO_2_/C_3_H_6_ selectivity. This offers a viable strategy for selective CO_2_ capture from multicomponent gas mixtures without compromising the intrinsic performance of zeolite 5A.

CO_2_ and C_3_H_6_ adsorption data are fitted with the Langmuir and Sips models to elucidate the underlying adsorption mechanism. As shown in Figures S11 and S12, the Sips model provides an excellent fit across all temperatures, whereas the Langmuir model (Figures S13 and S14) accurately represents the data only at 348 and 373 K. The Langmuir model, which assumes uniform, monolayer adsorption, is more applicable at high temperature, where increased molecular motion restricts the formation of multilayers. In contrast, the Sips model, accounting for surface heterogeneity and multilayer adsorption, fits better at 298 and 323 K, where surface heterogeneity plays a significant role. The evaluation parameters, including Adj. *R*^2^, maximum adsorption capacity (*q*ₘ), normalized standard deviation (Δ*Q*), and equilibrium constants (*k*_L_ for Langmuir model and *k*_S_ for Sips model), are listed in Tables S3–S6. These parameters demonstrate the superior performance of the Sips model, which yields higher Adj. *R*^2^ values and lower Δ*Q* than that with the Langmuir model.

Thermodynamic parameters (Δ*G*^0^, Δ*H*^0^, and Δ*S*^0^) [84] are evaluated to understand the adsorption behavior of CO_2_ and C_3_H_6_ on both pristine and modified zeolite 5A. Using Equations (1)–(3), the values of Δ*H*^0^ and Δ*S*^0^ are determined from the slope and intercept of the van’t Hoff plots (ln(*k*) vs. 1/*T*), and Δ*G*^0^ is calculated accordingly. The universal gas constant (R) is 8.314 J mol^−1^ K^−1^, and temperature is expressed in Kelvin. The equilibrium constant *k*_S_ used in the thermodynamic analysis is derived from the adsorption equilibrium constant obtained by fitting the Sips isotherm model (Equation (5)) to the experimental data at different temperatures. To ensure correct use of the logarithm in the van’t Hoff analysis, *k*_S_ is expressed as a dimensionless quantity by normalizing it with respect to the standard pressure.

(1) $${\Delta G}^{0}\;=\;-RT\;\ln(k)$$

(2) $${\Delta G}^{0}\;=\;{\Delta H}^{0}-T{\Delta S}^{0}$$

(3) $$\ln(k)=-\frac{{\Delta H}^{0}}{RT}+\frac{{\Delta S}^{0}}{R}$$

**Figure 8:**
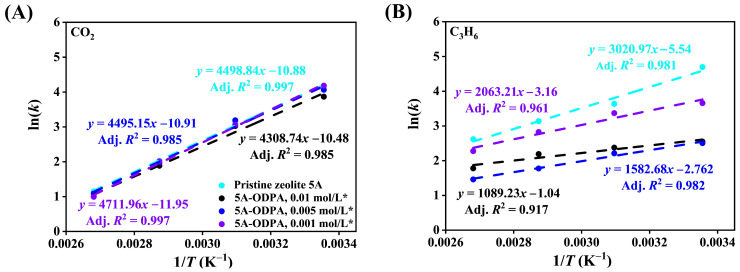
Van’t Hoff plots of ln(*k*) versus 1/*T* for (**A**) CO_2_ and (**B**) C_3_H_6_ adsorption in pristine and ODPA-modified zeolite 5A. Asterisk (*) denotes the THF-washed materials after modification.

Figure 8 shows the relationship between ln(*k*) and 1/*T* for CO_2_ and C_3_H_6_ for pristine and THF-washed ODPA-modified zeolite 5A, whereas Figure S15 shows the unwashed modified materials. The corresponding thermodynamic parameters derived from these plots are summarized in Table 2 and Table S7 for CO_2_ and C_3_H_6_, respectively. Overall, all materials exhibit thermodynamically favorable adsorption, as indicated by the negative values of Δ*G*^0^.

For pristine zeolite 5A, Δ*G*^0^ values for CO_2_ adsorption ranged from −10.42 to −3.63 kJ/mol, compared with −11.38 to −7.92 kJ/mol for C_3_H_6_. The corresponding Δ*H*^0^ values are −37.70 kJ/mol for CO_2_ and −25.12 kJ/mol for C_3_H_6_ over 298–373 K. The stronger dependence of CO_2_ adsorption enthalpy on coverage suggests more-pronounced energetic interactions at the cation sites, consistent with the interpretation proposed by Drenchev et al. [85].

These results confirm that adsorption is exothermic and follows an associative mechanism for both gases [86]. In the ODPA-modified zeolite 5A, the adsorption remains exothermic and spontaneous, as shown by the consistently negative Δ*G*^0^ values. For CO_2_, Δ*H*^0^ varies from −39.18 kJ/mol (0.001 mol/L ODPA-modified zeolite 5A with THF washed) to −30.70 kJ/mol (0.01 mol/L ODPA-modified zeolite 5A unwashed), with the most negative Δ*S*^0^ value (−96.30 J/mol·K) observed for 0.001 mol/L ODPA-modified zeolite 5A with THF washed. For C_3_H_6_, Δ*H*^0^ ranges from −9.06 kJ/mol (for 0.01 mol/L washed) to −26.25 kJ/mol (for 0.005 mol/L ODPA-modified zeolite 5A unwashed). These variations in Δ*H*^0^ and Δ*S*^0^ with ODPA concentration and washing treatment suggest that surface modification can effectively tune the adsorption behavior of zeolite 5A.

THF washing of ODPA-modified zeolite 5A significantly influences the adsorption thermodynamic of CO_2_ and C_3_H_6_ in distinct ways. For CO_2_, the removal of loosely bound ODPA residues reduces surface obstruction and restores access to high-energy adsorption sites within the zeolite framework. As a result, the washed sample exhibits more negative Δ*H*^0^ and Δ*S*^0^, reflecting stronger adsorption and greater molecular ordering.

In contrast, C_3_H_6_ adsorption in the unwashed samples is hindered, because residual ODPA remaining on the surface increases steric congestion. After washing, this hindrance is reduced, allowing C_3_H_6_ to reach the modified surface more efficiently. Although Δ*H*^0^ becomes slightly less negative due to the removal of weakly bound ODPA residues, the influence of ODPA modification remains clearly visible in the washed samples. Thus, THF washing enhances CO_2_ adsorption by improving access to strong adsorption sites, while for C_3_H_6_ it mainly reduces surface hindrance. These molecule-specific responses explain the opposite changes in thermodynamic parameters.

**Figure 9:**
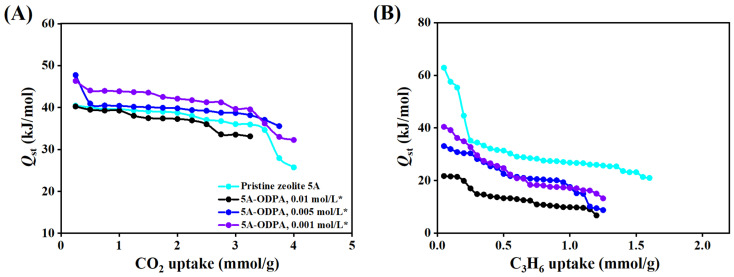
Isosteric adsorption heat of (**A**) CO_2_ and (**B**) C_3_H_6_ in pristine and the ODPA-modified zeolite 5A. Asterisk (*) denotes the THF-washed materials after modification.

Isosteric adsorption heat (*Q*_st_) of CO_2_ and C_3_H_6_, as presented in Figure 9 and Figure S16, provides a loading-dependent measure of adsorption energy. It should be noted that the van’t Hoff enthalpy (Δ*H*^0^) represents an averaged adsorption enthalpy derived from equilibrium constants over a finite temperature and coverage range, whereas the isosteric heat of adsorption (*Q*_st_) reflects coverage-dependent local adsorption energies. Consequently, quantitative differences between Δ*H*^0^ and *Q*_st_ are expected for energetically heterogeneous adsorbents such as zeolite 5A and its modified materials. The *Q*_st_ generally decreases with increasing CO_2_ and C_3_H_6_ capacity, reflecting the sequential occupation of adsorption sites with progressively lower binding energies. This decreasing phenomenon directly illustrates the transition from the strongest, most favorable adsorption sites to weaker adsorption sites as loading increases. For CO_2_ adsorption in pristine zeolite 5A (Figure 9A), *Q*_st_ ranges from 40.46 to 25.73 kJ/mol, whereas the ODPA-modified zeolite 5A material exhibits distinct behaviors depending on both the ODPA concentration and the washing treatment. For the washed materials, 0.01 mol/L ODPA-modified zeolite 5A exhibits a decrease in *Q*_st_ from 40.26 to 33.13 kJ/mol, the 0.005 mol/L ODPA-modified zeolite 5A material maintains relatively stable values between 47.75 and 35.56 kJ/mol, and the 0.001 mol/L ODPA-modified zeolite 5A material shows a moderate decrease from 46.35 to 32.27 kJ/mol. In contrast, the unwashed materials (Figure S16A) display different trends: 0.01 mol/L ODPA-modified zeolite 5A shows *Q*_st_ values decreasing from 36.20 to 26.21 kJ/mol, while the 0.005 and 0.001 mol/L ODPA-modified zeolite 5A materials start at high initial *Q*_st_ values of 42.20 and 43.01 kJ/mol and decrease to 26.78 and 25.83 kJ/mol, respectively, with increasing CO_2_ capacity. The *Q*_st_ of CO_2_ adsorption in ODPA-modified zeolite 5A varies with functionalization density. Low ODPA coverage (0.001 and 0.005 mol/L) increases *Q*_st_ relative to pristine zeolite due to unobstructed access to high-energy micropore sites, resulting in stronger CO_2_ adsorption. In contrast, at a higher ODPA coverage (0.01 mol/L), diffusion is hindered at the external surfaces, leading to slightly reduced adsorption and lower *Q_s_*_t_. These results indicate that ODPA modification primarily affects adsorption by imposing steric and diffusion barriers at the external surfaces of the modified zeolite 5A materials.

For C_3_H_6_ adsorption (Figure 9B), *Q*_st_ at zero coverage reaches 62.89 kJ/mol for pristine zeolite 5A, while it ranges from 40.40 to 6.71 kJ/mol for the washed ODPA-modified zeolite 5A materials and from 46.80 to 8.44 kJ/mol for the unwashed materials (Figure S16B). Specifically, for the unwashed 0.01 mol/L material, *Q*_st_ varies between 27.04 and 20.88 kJ/mol, indicating predominantly physisorption with relatively weak adsorbate—surface interactions, likely due to partially blocked high-energy adsorption sites caused by the high coverage of ODPA on the zeolite 5A surface after modification. Overall, *Q*_st_ values below 40 kJ/mol correspond to physisorption, whereas values above 40 kJ/mol suggest stronger interactions between the adsorbate and the modified surface.

Separation factor (*R*_L_) for CO_2_ and C_3_H_6_ adsorption in pristine and the ODPA-modified zeolite 5A materials at 298 K is shown in Figure S17. All materials, including pristine and modified zeolites, exhibit *R*_L_ values between 0 and 1, indicating favorable adsorption across all capacities of both gases. The results suggest that while pristine zeolite 5A displayed the best performance, the modified zeolites also demonstrated effective adsorption within the favorable *R*_L_ range, underscoring the positive effect of the modifications.

### 2.3. Adsorption Kinetics

**Figure 10:**
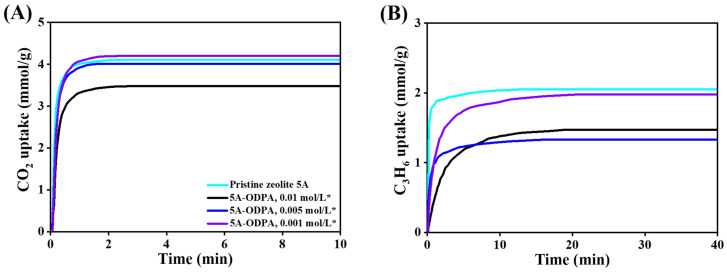
Time-dependent uptake of (**A**) CO_2_ and (**B**) C_3_H_6_ in pristine and the ODPA-modified zeolite 5A at 298 K and 100 kPa. Asterisk (*) denotes the THF-washed materials after modification.

Adsorption uptake of CO_2_ and C_3_H_6_ in pristine and the ODPA-modified zeolite 5A materials are investigated at 298, 323, 348, and 373 K under 100 kPa as shown in Figure 9 and Figures S18–S21. Across all temperatures, CO_2_ consistently exhibits faster adsorption uptake than C_3_H_6_, due to its smaller molecular size (3.3 Å vs. 3.9 Å for C_3_H_6_) [87]. At 298 K, as shown in Figure 10, pristine zeolite 5A demonstrates the most rapid uptake, reaching equilibrium within 1.0 min for CO_2_ (4.10 mmol/g) and 12.0 min for C_3_H_6_ (2.05 mmol/g). For the material modified 0.01 mol/L ODPA, the washed material achieves 3.77 mmol/g CO_2_ in 2 min (Figure 10A) and 1.47 mmol/g C_3_H_6_ in 20 min (Figure 10B), whereas the unwashed counterpart requires 4.0 min for CO_2_ (Figure S18A) and 35 min (Figure S18B) for C_3_H_6_ to reach 2.83 mmol/g and 0.99 mmol/g, respectively. This trend is maintained at 323 K (Figure S19), where the washed material continues to outperform the unwashed one in both adsorption rate and capacity. At higher temperatures (348 and 373 K; Figures S20 and S21), the adsorption rate decreases due to the exothermic nature of the process, but the relative performance order remains consistent: pristine > washed > unwashed.

Notably, at 0.005 mol/L ODPA and 298 K, the washed modified zeolite material nearly matches the pristine material in CO_2_ uptake, achieving 4.01 mmol/g within 1.0 min, while showing a moderate reduction in C_3_H_6_ uptake to 1.32 mmol/g in 20 min. This observation reinforces that proper surface modification followed by washing preserves rapid CO_2_ adsorption uptake and pore accessibility, offering an effective strategy to fine-tune selectivity and chemical compatibility for targeted gas separation application.

The adsorption behavior shows a clear concentration-dependent effect of ODPA modification. When adsorption uptake is expressed per total mass of the modified material, the apparent equilibrium uptake of C_3_H_6_ decreases with increasing ODPA coverage, and this reduction persists at high ODPA concentrations (0.01 and 0.005 mol/L) even after extended equilibration. This trend does not result from a loss of intrinsic adsorption sites within the zeolite framework; rather, ODPA grafting at the external surface restricts access to the adsorption sites. At a low ODPA concentration (0.001 mol/L), adsorption exhibits predominantly diffusion-limited behavior. ODPA slows the uptake rate but only weakly affects the equilibrium capacity, indicating that the pore entrances remain relatively open. At higher ODPA coverages, steric hindrance limits access of large molecules, which leads to a reduced apparent equilibrium uptake when adsorption is normalized by the total material mass. CO_2_ adsorption remains largely unchanged across all ODPA concentrations. Although both CO_2_ (3.3 Å) and C_3_H_6_ (3.9 Å) are smaller than the nominal pore aperture of zeolite 5A (~4.2 Å), grafted ODPA partially narrows the effective pore entrance. This narrowing preferentially impedes the large C_3_H_6_ molecule while still permitting unhindered access for CO_2_. Based on the mass of zeolite 5A, the intrinsic adsorption behavior can be clearly evaluated. As shown in Figure S22, for the 0.01 and 0.005 mol/L ODPA-washed zeolite 5A materials, expressing the C3H6 uptake in a zeolite 5A mass basis (excluding ODPA) shows that the equilibrium uptake remains essentially unchanged, whereas the uptake rate decreases significantly. This result demonstrates that ODPA modification does not reduce the intrinsic number of adsorption sites within zeolite 5A, but instead introduces a diffusion barrier at the external surfaces. Partial recovery of C_3_H_6_ uptake after THF washing, together with sharpening of the ^31^P NMR signal, confirms that ODPA does not bind within the micropores and primarily resides at external surfaces, creating a combined steric and diffusion barrier.

The localization and effect of ODPA on zeolite 5A are supported by complementary NMR, adsorption, and kinetic data. Solid-state ^31^P and ^13^C NMR spectra confirm that ODPA grafts onto the external surfaces, while THF washing removes physisorbed molecules and preserves a covalently bound monolayer. ^27^Al and ^29^Si NMR spectra show negligible framework distortion, indicating that ODPA functionalization occurs at the surface without affecting the intrinsic zeolite structure. Adsorption isotherms of CO_2_ and C_3_H_6_ show that CO_2_ uptake remains unchanged after ODPA modification, while C_3_H_6_ adsorption decreases at high ODPA coverage. These trends indicate restricted access at the pore entrances for larger molecules. Kinetic measurements further show that C_3_H_6_ adsorption slows in ODPA-modified zeolite 5A materials, while CO_2_ uptake remains rapid, consistent with a diffusion barrier created by surface-bound ODPA. Normalization of adsorption uptake to the zeolite mass demonstrates that intrinsic adsorption sites remain accessible. Collectively, these observations support the proposed steric-blocking mechanism, whereby ODPA primarily localizes at external surfaces, hindering larger molecules such as C_3_H_6_ while allowing smaller molecules like CO_2_ to diffuse freely.

#### 2.3.1. Ideal Selectivity

Ideal selectivity in this work is defined as the ratio of the adsorbed amounts of CO_2_ to C_3_H_6_ at the same adsorption time, obtained from single-component kinetic adsorption measurements. This metric therefore represents ideal kinetic selectivity, reflecting differences in diffusion and transport rates within the zeolite framework, rather than ideal selectivity derived from adsorption isotherms.

**Figure 11:**
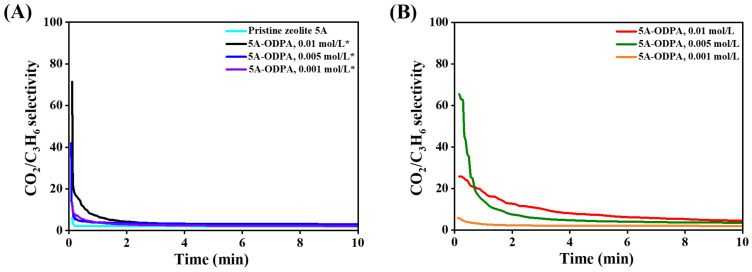
Ideal selectivity of CO_2_/C_3_H_6_ adsorption as the function of time in pristine and the ODPA-modified zeolite 5A at 298 K: (**A**) pristine and modified zeolite 5A materials (THF washed after modification) and (**B**) unwashed modified zeolite 5A. Asterisk (*) denotes the THF-washed materials after modification.

The CO_2_/C_3_H_6_ model system provides valuable guidance for designing adsorbents capable of addressing challenging separations involving chemically diverse molecules, such as CO_2_/olefins or polar/nonpolar mixtures. The impact of ODPA surface functionalization on CO_2_/C_3_H_6_ ideal selectivity at 298, 323, 348, and 373 K is shown in Figure 11 and Figure S23. ODPA coatings enhance selectivity, demonstrating their effectiveness in improving separation performance. Ideal selectivity of CO_2_ over C_3_H_6_ is consistently higher in ODPA-modified zeolite 5A than in pristine zeolite 5A, which exhibits a selectivity of 6.07, particularly during the initial adsorption phase.

At 298 K, as shown in Figure 11A, the washed 0.01 mol/L ODPA-modified zeolite 5A material shows the highest initial ideal selectivity, exceeding 73, before gradually stabilizing after about 4 min. In contrast, unwashed 0.005 and 0.01 mol/L ODPA-modified zeolite 5A materials (Figure 11B) have initial kinetic selectivities of approximately 65 and 25, respectively, reaching equilibrium after 6 and 9 min. The ranking of materials by ideal selectivity is as follows: 0.01 mol/L ODPA-modified zeolite 5A with THF washed ˃ 0.005 mol/L ODPA-modified zeolite 5A unwashed ˃ 0.005 mol/L ODPA-modified zeolite 5A with THF washed ˃ 0.01 mol/L ODPA-modified zeolite 5A unwashed > 0.001 mol/L ODPA-modified zeolite 5A with THF washed > 0.001 mol/L ODPA-modified zeolite 5A unwashed > pristine zeolite 5A. Notably, materials with high ideal selectivity take a long time to reach equilibrium, indicating significant diffusion differences between CO_2_ and C_3_H_6_.

These results indicate that ODPA functionalization effectively tunes zeolite surface transport properties, selectively tuning CO_2_ diffusion relative to C_3_H_6_. This enhancement benefits kinetic separations of molecules with similar sizes but differing mobilities. Furthermore, as shown in Figure S23A–C, ideal selectivity increases with temperature for all materials, suggesting that elevated temperatures amplify differential diffusion rates and improve separation efficiency.

We acknowledge that ideal selectivity derived from single-component kinetic measurements is an idealized metric and does not account for competitive adsorption or coupled diffusion in mixed-gas systems. While competitive adsorption may reduce absolute selectivity values, the relative ranking of materials and the observed enhancement in CO_2_/C_3_H_6_ kinetic discrimination remain valid, as they arise from diffusion contrasts introduced by ODPA functionalization. As further illustrated in Figure S23A–C, ideal selectivity increases with temperature for all materials, suggesting that elevated temperatures amplify differential diffusion rates and further enhance separation efficiency.

Overall, these results demonstrate that ODPA functionalization effectively tunes the surface transport properties of zeolite 5A, selectively enhancing CO_2_ diffusion relative to C_3_H_6_. While future mixed-gas diffusion or breakthrough experiments are needed to fully evaluate performance under practical conditions, the findings provide insight into selective diffusion and transport mechanisms, indicate the promising potential of ODPA-functionalized zeolite 5A for selective kinetic separations of light hydrocarbons and CO_2_, and offer guidance for designing adsorbents for challenging chemical separations.

#### 2.3.2. Kinetic Adsorption Analysis

**Figure 12:**
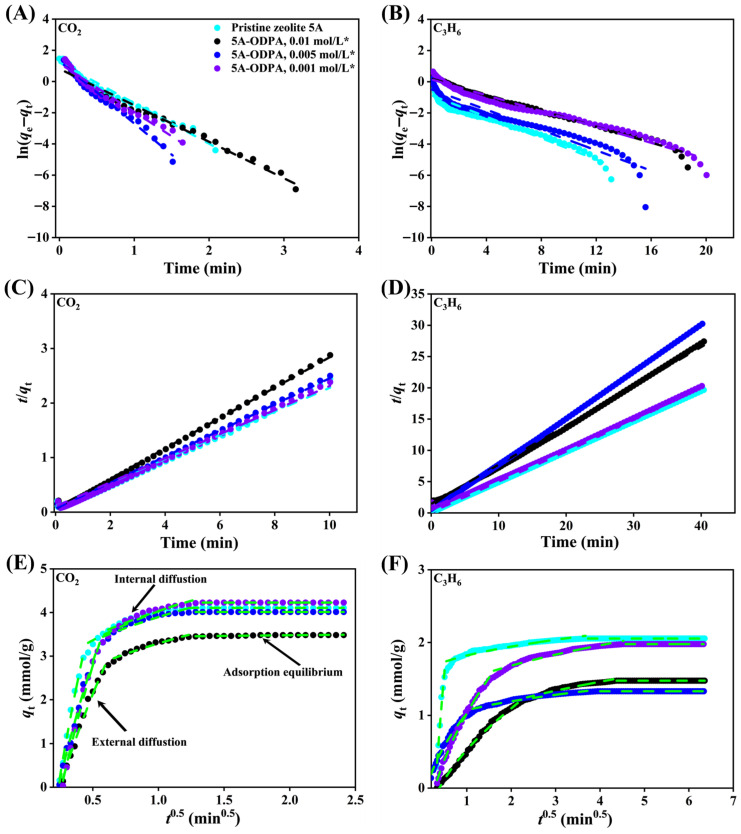
Kinetic model fitting for CO_2_ and C_3_H_6_ adsorption in pristine and the ODPA-modified zeolite 5A (washed with THF; noted with asterisk) at 298 K; (**A**,**B**) linear fitting of ln(*q*_e_ − *q*_t_) versus time using pseudo-first-order model for CO_2_ and C_3_H_6_, respectively, (**C**,**D**) linear fitting of *t*/*q*_t_ versus time using pseudo-second-order model for CO_2_ and C_3_H_6_, respectively, and (**E**,**F**) intraparticle diffusion model plots for CO_2_ and C_3_H_6_ adsorption, respectively.

Adsorption uptake of CO_2_ and C_3_H_6_ on both pristine and the ODPA-modified zeolite 5A materials at 298 K were investigated using pseudo-first-order and pseudo-second-order kinetic models. For the PFO model, the data are linearized by plotting ln(*q*_e_ − *q*_t_) versus time, where *q*_e_ and *q*_t_ represent the adsorption capacities at equilibrium and at time *t*, respectively. From the slopes and intercepts of these plots (Figure 12A,B) for pristine and the THF-washed ODPA-modified zeolite 5A materials and Figure S24 for unwashed modified materials), the PFO rate constant (*k*_1_) and the calculated equilibrium adsorption capacity (*q*_cal_) were obtained. The corresponding kinetic parameters for CO_2_ and C_3_H_6_ are summarized in Table 3 and Table 4, respectively.

Similarly, the PSO model is evaluated by plotting *t*/*q*_t_ versus time in Figure 12C,D for pristine and THF-washed ODPA-modified zeolite 5A materials and Figure S25 for unwashed modified materials, from which the PSO rate constant (*k*_2_) and *q*_cal_ are derived. The experimentally measured equilibrium adsorption capacities (*q*_exp_), which are listed in Table 3 and Table 4, were obtained by fitting both models to the kinetic data at 298 K.

By comparing the *q*_exp_ and *q*_cal_ (Table 3 and Table 4), the goodness of fit for each model is assessed, enabling the identification of the dominant adsorption mechanism for CO_2_ and C_3_H_6_ on pristine and ODPA-functionalized zeolite 5A. This analysis provides clear insight into the influences of ODPA modification on the adsorption uptake of zeolite 5A.

The application of PFO and PSO kinetic models for CO_2_ and C_3_H_6_ adsorption is physically justified by differences in molecular size and diffusion behavior in ODPA layers. CO_2_ (kinetic diameter: 3.3 Å) diffuses readily through the 4.2 Å pores of pristine zeolite 5A, resulting in adsorption kinetics primarily governed by external mass transfer and physisorption, which are well described by the PFO model. CO_2_, being a small molecule with minimal intraparticle diffusion resistance, rapidly migrates to accessible adsorption sites. In contrast, C_3_H_6_ (kinetic diameter: 3.9 Å) experiences greater steric hindrance even in unmodified zeolite, making its adsorption rate more dependent on diffusion through the pore openings, consistent with the PSO model. ODPA modification alters the surface chemistry, significantly affecting adsorption of both gases. The two main factors controlling adsorption in ODPA-modified zeolite 5A are the ODPA concentration and the use of a THF washing step, which together influence the balance between adsorption strength and diffusion resistance, shaping both capacity and kinetics. This strategy enables tuning not only for higher overall adsorption but also for enhanced CO_2_ selectivity over C_3_H_6_. These interpretations are consistent with the intraparticle diffusion analysis, which shows slower diffusion rates for C_3_H_6_ relative to CO_2_ and indicates the influence of ODPA modification on molecular diffusion.

For CO_2_ adsorption (Table 3), pristine zeolite 5A exhibits strong uptake and is well described by the PFO model, but PSO fits reveal that chemisorption-like interactions become more influential when ODPA is present. At mid-to-high ODPA levels (0.005–0.01 mol/L), PSO control becomes evident, with calculated capacities (*q*_cal_) closely matching or exceeding experimental data (*q*_exp_) and higher rate constants (*k*_2_). THF washing amplifies this effect by clearing blocked pores and exposing additional active sites, improving PSO fits and overall efficiency. This treatment preserves the inherent CO_2_ adsorption capacity by providing a modified surface environment that favors CO_2_ adsorption, while simultaneously reducing the adsorption of C_3_H_6_.

For C_3_H_6_ (Table 4), PSO kinetics model also fit well, suggesting chemisorption-driven uptake remains dominant even after modification. Pristine zeolite 5A shows fast kinetics, while ODPA treatment slightly reduces initial capacity predicted by first-order models but preserves strong PSO performance, especially after washing. THF washing consistently raises *q*_cal_ and *k*_2_, likely by removing surface blockages and lowering diffusion barriers. Although hydrocarbons exhibit somewhat weaker, more diffusion-limited uptake in modified materials, this reinforces the selective advantage for CO_2_ under chemisorption-dominant conditions.

Therefore, THF washing of the ODPA-modified zeolite 5A material improves their PSO behavior and enhances active-site accessibility. The most effective design pairs a mid-to-high ODPA dose with THF washing, producing high *q*_cal_ and *k*_2_ values that reflect fast, efficient, and selective chemisorption. This combination not only maximizes total CO_2_ uptake but also strengthens CO_2_ selectivity over hydrocarbons, offering a clear pathway for tailoring zeolite 5A toward more selective and responsive gas adsorption applications.

Intraparticle diffusion model analysis of CO_2_ and C_3_H_6_ adsorption in pristine and the ODPA-modified zeolite 5A materials at 298 K reveals a multistep diffusion mechanism. Adsorption proceeds through three distinct phases; external-film diffusion, intraparticle diffusion, and surface adsorption equilibrium, as shown in Figure 12E and Figure S26A for CO_2_, and Figure 12F and Figure S26B for C_3_H_6_. Boundaries between these phases are determined by plotting *q*_*t*_ versus *t*^0.5^, segmenting the curves based on changes in slope, and fitting each linear segment with the intraparticle diffusion model. The start and end points of each region are guided by the extent of linearity and supported by high adjusted coefficients of determination (Adj. *R*^2^), with intersections of the fitted lines defining the transitions between diffusion regimes. These phases exhibit decreasing diffusion rate constants (*K*_diff_), reflecting a transition from rapid mass transfer on the external surface to slow diffusion within the pores, followed by adsorption site saturation. The diffusion parameters for each phase are summarized in Table S8 for CO_2_ and Table S9 for C_3_H_6_.

For CO_2_ adsorption, the pristine zeolite 5A shows the highest initial diffusion rate (*K*_diff1_ = 18.21), indicating that CO_2_ molecules rapidly cross the external film with minimal resistance. This is followed by a slower intraparticle diffusion phase (*K*_diff2_ = 2.27), where molecular transport is controlled by movement through the internal pore structure of the zeolite. The final phase, corresponding to surface adsorption equilibrium, shows an almost negligible diffusion rate (*K*_diff3_ ≈ 6.47 × 10^−15^), signaling those active sites are saturated and equilibrium is reached.

Upon modification with ODPA, the adsorption behavior changes noticeably, and the initial external film diffusion rates for CO_2_ decrease compared to pristine 5A, with *K*_diff1_ values ranging roughly between 6.41 and 14.31, suggesting that surface modification introduces additional resistance to mass transfer. Likewise, intraparticle diffusion rates also decline, indicating that the ODPA molecules partially block the surface of zeolite, restricting CO_2_ movement inside the material. Despite these reductions, the surface equilibrium phase still shows near-zero diffusion rates, demonstrating that the saturation of adsorption sites is ultimately achieved, although the process may be slightly slower.

When examining C_3_H_6_ adsorption, a similar trend but with overall lower diffusion rates is observed, and the pristine zeolite 5A exhibits a moderate initial diffusion rate (*K*_diff1_ = 9.64), which already suggests slower external film diffusion for C_3_H_6_ compared to CO_2_. The intraparticle diffusion rate (*K*_diff2_ = 0.30) is significantly lower, highlighting substantial resistance within the pores. The equilibrium phase shows an almost zero diffusion rate (*K*_diff3_ ≈ 3.69 × 10^−16^), indicating saturation at the surface.

After ODPA modification, the initial diffusion rates for C_3_H_6_ drop sharply to values between 0.24 and 2.65, implying that molecular access to the external surface is significantly hindered. The intraparticle diffusion rates remain low but vary slightly, falling approximately between 0.28 and 0.70, suggesting that internal diffusion is still constrained but comparable or slightly improved over the pristine material in some cases. The equilibrium diffusion rates remain close to zero, confirming that saturation is reached in all scenarios.

Comparison between pristine and the ODPA-modified zeolite 5A materials indicates that surface modification with ODPA consistently slows down both the external film diffusion and the internal pore diffusion of CO_2_ and C_3_H_6_. The effect is more pronounced for C_3_H_6_, which experiences a stronger diffusion hindrance after modification. The gradual decrease in *K*_diff_ values from the first to the third phase supports the sequential nature of the adsorption process, transitioning from fast external diffusion to slower intraparticle diffusion and eventually equilibrium at the surface. This detailed kinetic analysis elucidates the effect of surface chemistry and pore environment on alterations in gas adsorption dynamics, providing useful insights for optimizing zeolite modifications tailored for specific gas separations or storage applications.

#### 2.3.3. Activation Energy for CO_2_ and C_3_H_6_ Adsorption

**Figure 13:**
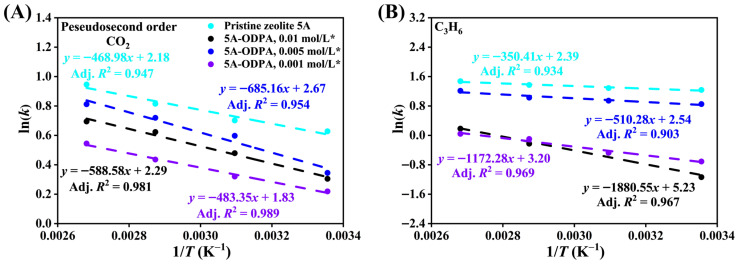
Arrhenius plots of ln(*k*) versus 1/*T* for pseudo-second-order kinetic model. (**A**) CO_2_ and (**B**) C_3_H_6_ adsorption in pristine and THF-washed modified zeolite 5A materials (noted with asterisk).

Activation energy values and kinetic behavior of CO_2_ and C_3_H_6_ adsorption in pristine and ODPA-modified zeolite 5A are analyzed using Arrhenius plots, with relation between the Natural Logarithm rate constant ln(*k*) and the inverse temperature (1/*T*), as shown in Figure 13 and Figures S27–S29. Figure 13A,B present the Arrhenius plots derived from the PSO kinetic model for CO_2_ and C_3_H_6_ adsorption in pristine A and the THF-washed modified zeolite 5 materials, respectively, while Figure S28A,B display the corresponding results for the unwashed modified materials. Likewise, Figure S27A,B show the PFO Arrhenius plots for CO_2_ and C_3_H_6_ adsorption in pristine and the THF-washed modified zeolite 5A materials; Figure S29A,B depict the equivalent plots for the unwashed materials. The slope of each fitted line is used to calculate the *E*_a_, and the results are summarized in Table 5, indicating the influence of ODPA modification, washing treatment, and gas type on the overall adsorption uptake.

For CO_2_ adsorption, pristine 5A exhibits relatively low activation energies in both PFO (1.49 kJ/mol) and PSO (3.90 kJ/mol) models. This indicates that the kinetics of CO_2_ adsorption in unmodified zeolite 5A proceed with modest energy barriers, reflecting efficient diffusion and interaction with adsorption sites. Upon ODPA modification, there is a general increase in activation energy values for CO_2_, with the highest values observed for the 0.01 mol/L modified materials (*E*_a_ up to 11.11 kJ/mol in PSO). This suggests that surface modification introduces additional energetic barriers, likely due to steric hindrance or altered surface chemistry that impedes rapid adsorption. However, the high coefficients of determination (Adj. *R*^2^ = 1) demonstrate that the kinetic data well-described by PSO models after modification.

C_3_H_6_ adsorption exhibits a stronger sensitivity to surface modification, with pristine 5A showing low activation energies (1.18 kJ/mol PFO, 2.91 kJ/mol PSO) similar to CO_2_ but with slightly lower energy barriers overall. The modified zeolite 5A, particularly at high ODPA concentration (0.01 mol/L), shows considerably elevated activation energies (up to 15.63 kJ/mol PSO), indicating that C_3_H_6_ adsorption is more affected by surface functionalization. This likely stems from increased diffusion resistance and decreased availability of active sites for olefin adsorption caused by ODPA layers.

THF washing decreases the activation energy of CO_2_ adsorption by removing weakly bound ODPA residues, reducing steric hindrance, and improving access to high-energy micropore sites, while the remaining ODPA preserves surface modification. For C_3_H_6_, although washing removes loosely bound fragments, the retained ODPA layer still modifies the external surface. Larger C_3_H_6_ molecules now penetrate deeper micropores, encountering additional confinement resistance, which increases the apparent activation energy. This behavior illustrates the combined effect of residual surface modification and micropore accessibility on adsorption uptake.

Comparing both gases, CO_2_ generally requires lower activation energy for adsorption in pristine and modified zeolite 5A, indicating its relatively easier access and stronger affinity to zeolite surface sites. In contrast, C_3_H_6_ adsorption is more kinetically hindered post-modification than CO_2_, consistent with its larger molecular size and sensitivity to pore blockage by ODPA.

Between modified materials, the activation energy trends do not strictly correlate with ODPA concentration, suggesting that factors such as washing treatment (* materials), molecular packing, and surface coverage heterogeneity also influence kinetic barriers. The analysis confirms that although surface modification with ODPA increases adsorption activation energy and introduces kinetic constraints for both gases, the adsorption processes retain Arrhenius-type behavior with clear temperature dependence. This provides a comprehensive kinetic framework to optimize zeolite modification for selective gas adsorption applications by balancing surface chemistry and diffusion properties.

To contextualize the operating conditions for industrial CO_2_/C_3_H_6_ separation, adsorption isotherms are obtained at multiple pressures up to 100 kPa, and adsorption kinetics are evaluated at 100 kPa on both pristine and ODPA-modified zeolite 5A using single-component CO_2_ and C_3_H_6_ gases. All samples undergo thermal activation under dry conditions prior to measurement. Under the tested conditions, 298 K and 100 kPa with 0.01 mol/L ODPA and THF washing maintain CO_2_ uptake in modified zeolite 5A at levels comparable to pristine zeolite 5A while reducing C_3_H_6_ uptake. THF washing removes physically adsorbed ODPA, which hinders diffusion through the chemically bound ODPA layer, thereby improving CO_2_ uptake kinetics. ODPA functionalization hinders C_3_H_6_ diffusion, demonstrating that surface modification kinetically controls gas transport. These findings are relevant to industrial CO_2_/C_3_H_6_ separation processes, where selective transport of CO_2_ over hydrocarbons is desirable. Limitations include the absence of mixture adsorption experiments, which future work can explore to fully assess industrial applicability.

## 3. Materials and Methods

### 3.1. Surface Modification of Zeolite 5A

Pristine zeolite 5A in powder form (Linde type, Ca*_n_*Na_12−2*n*_[(AlO_2_)_12_(SiO_2_)_12_] xH_2_O, <10 μm, Sigma-Aldrich, St. Louis, MO, USA, catalog number 233676) with a Ca^2+^/Na^+^ molar ratio of approximately 0.96, as determined from EDS-derived elemental compositions, together with tetrahydrofuran (C_4_H_8_O, 99.9%; MACKLIN Biochemical Co., Ltd., Shanghai, China, catalog no. T818769) and *n*-octadecylphosphonic acid (C_18_H_39_O_3_P, ≥97.0%; MACKLIN Biochemical Co., Ltd., Shanghai, China, catalog no. O836538), were used for the modification process without further purification. A total of 3.00 g of pristine zeolite 5A was thermally treated in air at 573 K for 4 h and then cooled to room temperature. A weight loss of approximately 17% was observed, attributed to dehydration and volatile removal, resulting in ~2.49 g of activated zeolite. This mass was subsequently divided into three portions of approximately 830 mg each for further modification. Each portion was dispersed in 200 mL of tetrahydrofuran (THF) containing ODPA at concentrations of 0.01, 0.005, and 0.001 mol/L, respectively. Suspensions were stirred and allowed to soak at ambient temperature for 24 h to facilitate surface functionalization. After the soaking, the mixtures were filtered, and the resulting solid residues (filter cakes) were collected. Each filter cake was transferred to a Teflon-lined stainless-steel autoclave, sealed, and subjected to hydrothermal treatment at 393 K for 12 h. After cooling to room temperature, each modified sample was divided into two fractions. One was retained unwashed, while the other was washed four times with fresh THF. Both sets of samples were dried in a vacuum oven at room temperature until constant mass was achieved. A schematic overview of the modification procedure is presented in Scheme S1.

### 3.2. Characterization of Pristine and Modified Zeolite 5A

Structure, chemical, and thermal properties of pristine and the ODPA-modified zeolite 5A materials were characterized using a combination of analytical techniques. X-ray diffraction (XRD) was performed using an Ultima IV multi-functional diffractometer (Rigaku, Tokyo, Japan) with Cu-Kα radiation (λ = 1.542 Å) at 50 kV and 40 mA, over a 2θ range of 5–80° at a scanning speed of 2°/min to confirm crystallographic integrity and detect phase changes. Fourier transform infrared spectroscopy (FTIR) analysis was conducted using a Thermo Fisher Nicolet iS50 spectrometer (Thermo Fisher Scientific, Waltham, MA, USA) within the 400–4000 cm^−1^ via the KBr pellet method, where 1–2 mg of sample mixed with 100–200 mg of KBr was pressed into a transparent pellet and scanned at 4 cm^−1^ resolution. A background correction with pure KBr was applied, and data was processed using Omnic software 9.2 for peak identification. Moisture control and fine grinding ensured minimal spectral interference. Surface morphology was examined using scanning electron microscopy (SEM; Tescan TS 5130MM, Tescan, Czech Republic), while elemental composition was analyzed using energy-dispersive X-ray spectroscopy (EDS; Oxford Instruments, Abingdon, UK, 50 mm^2^ detector). Surface chemical states were analyzed using X-ray photoelectron spectroscopy (XPS; Kratos Axis Ultra DLD-600W, Kratos Analytical Ltd., Manchester, United Kingdom), to confirm the presence and bonding of modifying agents. To evaluate thermal behavior, the thermal gravimetric analysis (TG) and differential scanning calorimetry (DSC) were conducted simultaneously using a Mettler Toledo TGA2 system under a nitrogen atmosphere from 25 to 1000 °C and at a heating rate of 10 °C/min. Solid-state nuclear magnetic resonance (NMR) spectroscopy analysis was performed to investigate the local structural environments of both pristine and 0.01 mol/L ODPA-modified zeolite 5A. Multinuclear magic-angle spinning (MAS) NMR spectra (^27^Al, ^29^Si, ^31^P, ^13^C) were acquired on a Bruker Avance III spectrometer (Bruker BioSpin, Rheinstetten, Germany) using a MAS probe at spinning rates of 8–12 kHz. Resonance frequencies were approximately 104.2 MHz (^27^Al), 79.5 MHz (^29^Si), 121.5 MHz (^31^P), and 100.6 MHz (^13^C), with chemical shifts referenced to 1.0 mol/L Al(NO_3_)_3_ (^27^Al), tetramethylsilane (29Si, ^13^C), and 85% H_3_PO_4_ (^31^P).

### 3.3. Adsorption Analysis

High-purity CO_2_ (≥99.999%) and propylene (C_3_H_6_ ≥ 99.5%), supplied by Qingdao Deyi Gas Co., Ltd. (Qingdao, China), were used. Single-component adsorption isotherms for both gases were measured using an automatic Sievert-type apparatus (PCTPro-2000, Setaram Instrumentation, Caluire et Cuire, France). Before breakthrough testing, all zeolite samples were subjected to thermal activation at 523 K for 2 h to eliminate residual water and strongly adsorbed contaminants from the pore structure. Adsorption measurements were performed on samples of pristine and ODPA-modified zeolite 5A samples, with ODPA concentrations of 0.01, 0.005, and 0.001 mol/L. Measurements were performed on modified samples divided into two trials, one with THF washing and the other without washing after modification. Adsorption isotherms were measured at temperatures of 298, 323, 348, and 373 K over a range of equilibrium pressures up to 100 kPa. Additionally, adsorption kinetics experiments were conducted at a constant pressure of 100 kPa across the same temperature range to evaluate the adsorption rate under fixed conditions.

Langmuir and Sips isotherm models were employed to interpret adsorption behavior. The Langmuir model (Equation (4)) assumes monolayer adsorption onto a homogeneous surface with a finite number of identical, energetically equivalent sites. In contrast, the Sips model (Equation (5)) combines features of the Langmuir and Freundlich isotherm model, accounting for heterogeneous multilayer adsorption.

(4) $$\frac{q}{q_{m}}=\frac{k_{L}p}{1+kp}$$

(5) $$\frac{q}{q_{m}}\;=\frac{{\left(k_{S}p\right)}^{n}}{1+{\left(kp\right)}^{n}}$$

where *k*_L_ and *k*_S_ denotes the normalized equilibrium constants for the Langmuir and Sips adsorption models, respectively; *p* represents the pressure exerted by the adsorbed molecules in kPa, while *n* serves as a parameter characterizing both the heterogeneity of the adsorption surface and the degree of favorability of the adsorption process [88].

Additionally, in Equation (6) a normalized standard deviation (Δ*Q*) was computed based on the residuals between the experimental data points (*q*_actual_) and the values predicted (*q*_predicted_) by the adsorption models. Furthermore, the adjusted coefficient of determination (Adj. *R*^2^) in Equation (7) was also determined [89].

(6) $$\Delta q=\sqrt{\frac{\sum_{i}^{n}{\left(q_{\mathrm{actual}}-\frac{q_{\mathrm{predicted}}}{q_{\mathrm{actual}}}\right)}^{2}}{n-1}}$$

(7) $$\mathrm{Adj}.\;R^{2}=1-\frac{(1-R^{2})(n-1)}{n-k-1}$$

where *n* is the number of observations, and *k* is the number of independent variables (predictors).

Separation factor (*R*_L_), calculated using Equation (8), is a key parameter in isotherm models that reflects the nature of the adsorption process. 0 < *R*_L_ < 1 denotes favorable adsorption, *R*_L_ = 1 indicates linear adsorption (no adsorption process is taking place), *R*_L_ > 1 suggests unfavorable adsorption, and *R*_L_ = 0 signifies irreversible adsorption [90].

(8) $$R_{L\;}=\frac{1}{1+kC_{0}}$$

where *k* represents the adsorption equilibrium constant of the isotherm model and *C*_0_ denotes the initial concentration of the adsorbate.

Isosteric adsorption heat (*Q*_st_) is determined by applying the Clausius–Clapeyron equation to the thermodynamic adsorption equilibrium relationship (Equation (9)), which connects the logarithmic of normalized pressure dependence to the standard adsorption enthalpy change (Δ*H*^0^) and entropy change (Δ*S*^0^) through temperature variation at specific coverage [91].

(9) $$\ln(p)=-\frac{\Delta H^{0}}{RT}+\frac{\Delta S^{0}}{R}\;$$

where R represents the universal gas constant, *T* denotes the temperature, and ∆*S*^0^ stands for the standard entropy of adsorption.

Isosteric adsorption heat is derived by differentiating the logarithm of normalized pressure with respect to temperature, as expressed in Equation (10).

(10) $$Q_{\mathrm{st}}={\left(\frac{\text{∂}\ln\left(p\right)}{\text{∂}T}\right)}_{\Gamma }\;RT^{2}$$

where *Q*_st_ represents the isosteric adsorption heat, indicating the energy required to remove one mole of adsorbed molecules from the surface of the adsorbent at a constant temperature, and *Γ* is specific surface coverage. This parameter is instrumental in understanding the nature of the interaction between the adsorbate and the adsorbent.

### 3.4. Kinetics Analysis

This study conducts a detailed analysis of adsorption uptake to evaluate the effectiveness of adsorption processes and identify potential rate-limiting steps. Three kinetic models, the pseudo-first-order, pseudo-second-order, and intra-particle diffusion models were applied to experimental data collected at a constant pressure of 100 kPa and a temperature of 298 K. These models, represented by Equations (11)–(13), describe different aspects of the adsorption process [92,93].

(11) $$\ln(q_{e}\;-\;q_{t})=\ln q_{e}\;-\;k_{1}t$$

(12) $$\frac{t}{q_{t}}=\frac{1}{k_{2}q_{e}^{2}}+\frac{t}{q_{e}}$$

(13) $$q_{t}=K_{\mathrm{diff}}\;\text{×}\;t^{0.5}+C$$

where *q*_e_ and *q*_t_ represent the normalized amounts of molecules adsorbed at equilibrium and at a specific time *t*, respectively—both expressed in mmol/g. The rate constants for the kinetic models are denoted as *k*_1_(min^−1^) for the pseudo-first-order model, *k*_2_ (g/mmol·min) for the pseudo-second-order model, and *K*_diff_ (mmol/g·min^1/2^) for the intra-particle diffusion model. The constant *C* corresponds to the thickness of the boundary layer surrounding the adsorbent particles.

Pseudo-first-order model, expressed by Equation (11), assumes that the rate of adsorption is proportional to the number of unoccupied sites on the adsorbent. This model is typically applicable when the adsorption rate depends mainly on the concentration of the adsorbate and is often associated with physical adsorption processes. In contrast, the pseudo-second-order model, given by Equation (12), assumes that the adsorption rate follows second-order kinetics. This implies that chemisorption, involving electron sharing or exchange between adsorbate and adsorbent, is the rate-limiting step. This model often provides a better fit for systems where strong interactions dominate the adsorption mechanism. Intra-particle diffusion model, represented by Equation (13), investigates diffusion of adsorbate molecules within the pores of the adsorbent as a possible rate-controlling step. This model helps to discern whether the adsorption process is limited by the movement of molecules inside the porous structure of the adsorbent rather than by surface interactions alone.

Activation energy for adsorption uptake can be calculated by combining kinetic models, such as the pseudo-first-order or pseudo-second-order models, with the Arrhenius equation. The rate constants (*k*_1_ or *k*_2_) derived from these models at different temperatures are used in the Arrhenius equation (Equation (14)).

(14) $$\ln(k)\;=\ln(A)-\frac{E_{a}}{RT}$$

where *k* is the rate constant, *A* is the pre-exponential factor, *E_a_* is the activation energy, R is the universal gas constant, and *T* is the absolute temperature.

By plotting ln(*k*) against 1/*T*, a linear relationship is obtained with slope −*E*_a_/*R*, from which the *E_a_* can be calculated. This approach allows determination of whether the adsorption process is controlled by strong or physical interactions, as indicated by the magnitude of the *E_a_*. It thus provides essential insight into the adsorption mechanism and the efficiency of adsorbent materials.

## 4. Conclusions

The surface modification of zeolite 5A with *n*-octadecylphosphonic acid (ODPA) can enhance the adsorptive separation of CO_2_ over C_3_H_6_ due to changes in surface chemistry. The zeolite remains its crystalline structure after ODPA modification and THF washing. The adsorption performance in both pristine and ODPA-modified zeolite 5A is well described by Sips isotherm model, with a higher accuracy than the Langmuir model. Thermodynamic analysis, with favorable Gibbs free energy and exothermic enthalpy changes, indicates that CO_2_ and C_3_H_6_ adsorption in pristine zeolite 5A is spontaneous. The isosteric adsorption heat is high in the ODPA-modified zeolite 5A, especially at low ODPA concentrations, indicating strong interaction between the gas molecules and the zeolite framework. The surface modification increases the diffusion resistance of CO_2_ and C_3_H_6_ into the zeolite, shifting the rate-limiting step from internal diffusion to surface penetration. Variations in ODPA concentration and THF washing significantly affect adsorption uptake and activation energy (*E*_a_). High ODPA concentration leads to increased *E*_a_ value, suggesting strong resistance to surface diffusion and restricting internal diffusion for gas molecules. THF washing reduces *E*_a_ value, particularly at high ODPA concentration, by removing weakly bound ODPA. These findings indicate the critical role of surface modification and post-synthesis treatment, such as ODPA modification and THF washing, in optimizing adsorption uptake and selectivity. The results provide a strong foundation for developing efficient adsorbents for CO_2_ capture and related applications.

## Figures and Tables

**Table 1:** Elemental composition (wt%) of pristine and ODPA-modified zeolite 5A obtained from SEM-EDS analysis. Asterisk (*) denotes THF-washed materials.

Material	O (wt%)	C (wt%)	Al (wt%)	Si (wt%)	Na (wt%)	Ca (wt%)	P (wt%)
Pristine zeolite 5A	52.08	0.00	16.35	18.37	4.94	8.26	0.00
5A-ODPA, 0.01 mol/L *	44.70	26.67	9.42	9.69	3.39	4.76	1.37
5A-ODPA, 0.01 mol/L	40.93	32.09	8.46	8.47	2.77	4.58	2.70
5A-ODPA, 0.005 mol/L *	45.12	19.51	12.13	12.55	3.29	6.56	0.84
5A-ODPA, 0.005 mol/L	43.36	29.42	9.34	9.22	2.85	4.86	1.22
5A-ODPA, 0.001 mol/L *	48.53	16.40	12.22	12.58	3.55	6.50	0.22
5A-ODPA, 0.001 mol/L	44.72	24.20	10.53	10.90	3.09	5.62	0.94

**Table 2:** Thermodynamic parameters of CO_2_ adsorption in pristine and the ODPA-modified zeolite 5A materials. Asterisk (*) denotes the THF-washed materials.

Material	Δ*H*^0^ (kJ/mol)	Δ*S*^0^ (J/mol·K)	Δ*G*^0^ (kJ/mol)			
			298 K	323 K	348 K	373 K
Pristine zeolite 5A	−37.40	−90.50	−10.42	−8.16	−5.90	−3.63
5A-ODPA, 0.01 mol/L *	−35.82	−87.20	−9.84	−7.66	−5.48	−3.30
5A-ODPA, 0.01 mol/L	−30.70	−72.80	−9.00	−7.18	−5.36	−3.54
5A-ODPA, 0.005 mol/L *	−37.37	−90.70	−10.33	−8.06	−5.80	−3.53
5A-ODPA, 0.005 mol/L	−36.05	−88.00	−9.81	−7.61	−5.41	−3.21
5A-ODPA, 0.001 mol/L *	−39.18	−96.30	−10.48	−8.07	−5.67	−3.26
5A-ODPA, 0.001 mol/L	−35.70	−86.50	−9.93	−7.77	−5.61	−3.45

**Table 3:** Kinetic parameters obtained from pseudo-first-order and pseudo-second-order models for CO_2_ adsorption in pristine and ODPA-modified zeolite 5A at the different ODPA concentrations (with and without THF washing). Asterisks (*) indicate THF-washed materials.

Material	Pseudo-First-Order (PFO)				Pseudo-Second-Order (PSO)			
	*q*_exp_	*q*_cal_	*k*_1_ (min^−1^)	Adj. *R*^2^	*q*_exp_	*q*_cal_	*k*_2_ (g/mmol·min)	Adj. *R*^2^
	(mmol/g)				(mmol/g)			
Pristine zeolite 5A	4.10	3.39	2.60	0.978	4.30	4.61	0.42	0.818
5A-ODPA, 0.01 mol/L *	3.48	2.26	2.33	0.982	3.71	3.59	1.38	0.992
5A-ODPA, 0.01 mol/L	2.79	1.41	0.81	0.904	3.06	3.11	0.50	0.927
5A-ODPA, 0.005 mol/L *	4.03	3.69	3.99	0.980	4.02	4.19	0.88	0.964
5A-ODPA, 0.005 mol/L	3.64	1.78	0.91	0.902	3.89	3.78	1.20	0.994
5A-ODPA, 0.001 mol/L *	4.22	3.20	3.11	0.964	4.48	4.53	0.45	0.849
5A-ODPA, 0.001 mol/L	3.71	2.39	2.18	0.962	3.98	3.95	0.61	0.956

**Table 4:** Kinetic parameters obtained from pseudo-first-order and pseudo-second-order models for C_3_H_6_ adsorption in pristine and ODPA-modified zeolite 5A at the different ODPA concentrations (with and without THF washing). Asterisks (*) indicate THF-washed materials.

Material	Pseudo-First-Order (PFO)				Pseudo-Second-Order (PSO)			
	*q*_exp_	*q*_cal_	*k*_1_ (min^−1^)	Adj. *R*^2^	*q*_exp_	*q*_cal_	*k*_2_ (g/mmol·min)	Adj. *R*^2^
	(mmol/g)				(mmol/g)			
Pristine zeolite 5A	1.99	0.58	0.34	0.919	2.07	2.06	3.23	1.000
5A-ODPA, 0.01 mol/L *	1.44	1.23	0.26	0.987	1.66	1.60	0.27	0.995
5A-ODPA, 0.01 mol/L	1.02	1.13	0.12	0.943	1.33	1.27	0.08	1.000
5A-ODPA, 0.005 mol/L *	1.25	1.19	0.33	0.922	1.33	1.34	2.15	1.000
5A-ODPA, 0.005 mol/L	1.17	1.09	0.19	0.973	1.38	1.40	0.14	0.991
5A-ODPA, 0.001 mol/L *	1.90	1.16	0.42	0.968	2.07	2.04	0.89	1.000
5A-ODPA, 0.001 mol/L	1.25	1.04	0.25	0.957	2.02	1.91	0.53	0.999

**Table 5:** Activation energies (*E*_a_) and coefficients of determination (Adj. *R*^2^) derived from Arrhenius plots for CO_2_ and C_3_H_6_ adsorption in pristine and modified zeolite 5A, based on pseudo-first-order and pseudo-second-order kinetic models. Asterisks (*) indicate THF-washed materials.

Material	*E*_a_ (kJ/mol)			
	Pseudo-First-Order		Pseudo-Second-Order	
	CO_2_	C_3_H_6_	CO_2_	C_3_H_6_
Pristine zeolite 5A	1.49	1.18	3.90	2.91
5A-ODPA, 0.01 mol/L *	2.32	9.36	4.89	15.63
5A-ODPA, 0.01 mol/L	5.18	7.56	11.11	12.62
5A-ODPA, 0.005 mol/L *	1.74	5.82	5.69	4.24
5A-ODPA, 0.005 mol/L	0.84	2.75	7.44	3.43
5A-ODPA, 0.001 mol/L *	1.72	2.67	4.02	9.74
5A-ODPA, 0.001 mol/L	0.60	3.22	7.34	5.48

## Data Availability

The raw data supporting the conclusions of this article will be made available by the authors upon request.

## Supplementary Materials

The following supporting information can be downloaded at: https://www.mdpi.com/article/10.3390/molecules31030564/s1, Scheme S1: Schematic flow diagram depicting the surface modification of zeolite 5A with *n*-octadecylphosphonic acid; Figure S1: FTIR spectra of 0.01 mol/L ODPA-modified zeolite 5A (red line), 0.005 mol/L ODPA-modified zeolite 5A (olive line), and 0.001 mol/L ODPA-modified zeolite 5A (dark orange line) after modification without THF washing; Figure S2: TG, and DSC curves of modified zeolite 5A with different concentrations and without THF washing after modification process: (A) modified zeolite 5A with 001 M of ODPA, (B) 0.005 mol/L ODPA-modified zeolite 5A, and (C) 0.001 mol/L ODPA-modified zeolite 5A; Figure S3: EDS spectra and elemental compositions of pristine and THF-washed modified zeolite 5A materials: (A) pristine zeolite 5A, (B) 5A-ODPA, 0.01 mol/L, (C) 0.005 mol/L ODPA-modified zeolite 5A, and (D) 0.001 mol/L ODPA-modified zeolite 5A; Figure S4: EDS spectra and elemental compositions of modified zeolite 5A without THF washing after modification process: (A) 0.01 mol/L ODPA-modified zeolite 5A, (B) 0.005 mol/L ODPA-modified zeolite 5A, and (C) 0.001 mol/L ODPA-modified zeolite 5A; Figure S5: Mapping distribution for different elemental compositions of modified zeolite 5A with THF washing after the modification process of pristine and modified zeolite 5A: (A) pristine zeolite 5A, (B) 0.01 mol/L ODPA-modified zeolite 5A, (C) 0.005 mol/L ODPA-modified zeolite 5A, and (D) 0.001 mol/L ODPA-modified zeolite 5A; Figure S6: Mapping distribution for different elemental composition of modified zeolite 5A without THF washing after modification process: (A) 0.01 mol/L ODPA-modified zeolite 5A, (B) 0.005 mol/L ODPA-modified zeolite 5A, and (C) 0.001 mol/L ODPA-modified zeolite 5A; Figure S7: Adsorption isotherms of (A) CO_2_ and (B) C_3_H_6_ in pristine and the ODPA-modified zeolite 5A materials at 298 K with different ODPA concentrations without washing after modification; Figure S8: adsorption isotherms of (A) CO_2_ and (B) C_3_H_6_ in pristine and ODPA-modified zeolite 5A materials measured at 323 K; Impact of ODPA concentration and washing Procedure. The materials washed with tetrahydrofuran after modification are marked with an asterisk (*); Figure S9: adsorption isotherms of (A) CO_2_ and (B) C_3_H_6_ in pristine and the ODPA-modified zeolite 5A materials measured at 348 K; Impact of ODPA concentration and washing Procedure; Figure S10: adsorption isotherms of (A) CO_2_ and (B) C_3_H_6_ in pristine and the ODPA-modified zeolite 5A materials measured at 373 K; Impact of ODPA concentration and washing Procedure; Figure S11: CO_2_ adsorption data fitted with the sips isotherm model across all temperatures: (A) 298 K, (B) 323 K, (C) 348 K, and (D) 373 K, reflecting the heterogeneous nature of adsorption sites in pristine and modified zeolite 5A; Figure S12: C_3_H_6_ adsorption data fitted with the sips isotherm model across all temperatures: (A) 298 K, (B) 323 K, (C) 348 K, and (D) 373 K, reflecting the heterogeneous nature of adsorption sites in pristine and modified zeolite 5A; Figure S13: CO_2_ adsorption data fitted with the Langmuir isotherm model across all temperatures: (A) 298 K, (B) 323 K, (C) 348 K, and (D) 373 K; Figure S14: C_3_H_6_ adsorption data fitted with the Langmuir isotherm model across all temperatures: (A) 298 K, (B) 323 K, (C) 348 K, and (D) 373 K; Figure S15: Van’t Hoff plots of ln(*k*) versus 1/*T* for (A) CO_2_ and (B) C_3_H_6_ adsorption in pristine and the ODPA-modified zeolite 5A materials without THF washing after modification for modified materials; Figure S16: Isosteric adsorption heat of (A) CO_2_ and (B) C_3_H_6_ in pristine and the ODPA-modified zeolite 5A materials without THF washing after modification for modified materials; Figure S17: Separation factor (*R*_L_) vs. initial concentration (*C*_0_) for adsorption in pristine and the ODPA-modified zeolite 5A materials at 298 K: (A) CO_2_ and (B) C_3_H_6_; Figure S18: Time-dependent uptake of (A) CO_2_ and (B) C_3_H_6_ in pristine and the ODPA-modified zeolite 5A materials at 298 K with different ODPA concentrations without washing after modification; Figure S19: Time-dependent uptake of (A) CO_2_ and (B) C_3_H_6_ in pristine and the ODPA-modified zeolite 5A materials at 323 K and 100 kPa; Figure S20: Time-dependent uptake of (A) CO_2_ and (B) C_3_H_6_ in pristine and the ODPA-modified zeolite 5A materials at 348 K and 100 kPa; Figure S21: Time-dependent uptake of (A) CO_2_ and (B) C_3_H_6_ in pristine and the ODPA-modified zeolite 5A materials at 373 K and 100 kPa; Figure S22: Time-dependent C_3_H_6_ uptake in pristine zeolite 5A, 0.005 mol/L ODPA-modified zeolite 5A (THF-washed), and 0.01 mol/L ODPA-modified zeolite 5A (THF-washed), based on zeolite 5A mass; showing unchanged equilibrium uptake and reduced adsorption rate after ODPA modification; Figure S23: Ideal selectivity of CO_2_/C_3_H_6_ adsorption in pristine and the ODPA-modified zeolite 5A materials at varying temperatures: (A) 323 K, (B) 348 K, (C), and 373 K; Figure S24: Linear fitting of ln(*q*_e_ − *q*_t_) versus time for the adsorption uptake in pristine and ODPA-modified zeolite 5A without THF washing after modification at 298 K using the PFO model: (A) CO_2_ and (B) C_3_H_6_; Figure S25: Linear fitting of *t*/*q*_t_ versus time for the adsorption uptake in pristine and ODPA-modified zeolite 5A without THF washing after modification at 298 K using the PSO model: (A) CO_2_ and (B) C_3_H_6_; Figure S26: Intraparticle diffusion model plots for (A) CO_2_ and (B) C_3_H_6_ adsorption in pristine and ODPA-modified zeolite 5A without THF washing after modification at 298 K; Figure S27: Arrhenius plots of ln(*k*) versus 1/*T* for PFO kinetic model. (A) CO_2_ and (B) C_3_H_6_ adsorption in pristine and the ODPA-modified zeolite 5A materials (washed with THF); Figure S28: Arrhenius plots of ln(*k*) versus 1/*T* for the PSO kinetic model. (A) CO_2_ and (B) C_3_H_6_ adsorption in pristine and the ODPA-modified zeolite 5A materials without THF washing after modification; Figure S29: Arrhenius plots of ln(*k*) versus 1/*T* for the PFO kinetic model. (A) CO_2_ and (B) C_3_H_6_ adsorption in pristine and the ODPA-modified zeolite 5A materials without THF washing after modification; Table S1: Thermal Analysis Data for pristine and ODPA-modified zeolite 5A; Table S2: Atomic composition (at%) of pristine and the ODPA-modified zeolite 5A materials from XPS analysis; Table S3: Adsorption isotherms parameters of the Sips model for CO_2_ adsorption in pristine and modified 5A zeolite. (The asterisk (*) denotes THF-washed materials.); Table S4: Adsorption isotherms parameters of the Sips model for C_3_H_6_ adsorption in pristine and modified 5A zeolite; Table S5: Adsorption isotherms parameters of the Langmuir model for CO_2_ adsorption in pristine and modified 5A zeolite; Table S6: Adsorption isotherms parameters of the Langmuir model for C_3_H_6_ adsorption in pristine and modified 5A zeolite; Table S7: Thermodynamic parameters for C_3_H_6_ adsorption in pristine and modified zeolite 5A; Table S8: The diffusion parameters for CO_2_ adsorption in pristine and the ODPA-modified zeolite 5A materials at 298 K; Table S9: The diffusion parameters for C_3_H_6_ adsorption in pristine and the ODPA-modified zeolite 5A materials at 298 K.

## Abbreviations

The following abbreviations are used in this manuscript:

ODPA	*n*-octadecylphosphonic acid
THF	Tetrahydrofuran
MOFs	Metal–organic frameworks
XRD	X-ray diffraction
FTIR	Fourier transform infrared spectroscopy
SEM	Scanning electron microscope
EDS	Energy-dispersive X-ray spectroscopy
XPS	X-ray photoelectron spectroscopy
TG	Thermal gravimetric analysis
DSC	Differential scanning calorimetry
*E*_a_	Activation energy
