# Supplementary material for: Effect of n-Octadecylphosphonic Acid Coating on Zeolite 5A for Adsorptive Separation of Carbon Dioxide and Propylene
Source: Molecules. 2026 Feb 5;31(3):564. doi: 10.3390/molecules31030564 (PMC12899014; doi:10.3390/molecules31030564)
Supplement: Supplementary file 1 [file molecules-31-00564-s001.zip › molecules-4119539-supplementary.pdf]

Effect of *n*-Octadecylphosphonic Acid Coating on Zeolite 5A for Adsorptive Separation of Carbon Dioxide and Propylene

Magdy Abdelghany Elsayed [1,3], Shixue Zhou [2,] *, Chengdong Zhang [1], Kun Zhang [1]

[1] *College of Energy and Mining Engineering, Shandong University of Science and Technology, Qingdao 266590, China*

[2] *College of Chemical and Biological Engineering, Shandong University of Science and Technology, Qingdao 266590, China*

[3] *Department of Mining and Petroleum Engineering, Faculty of Engineering, Al-Azhar University, Cairo 11884*

* Corresponding author.

E-mail address: zhoushixue66@163.com (Shixue Zhou).

Supporting Figures

Scheme S1. Schematic flow diagram depicting the surface modification of zeolite 5A with *n*-octadecylphosphonic acid.

Figure S1. FTIR spectra of 0.01 mol/L ODPA-modified zeolite 5A (red line), 0.005 mol/L ODPA-modified zeolite 5A (olive line), and 0.001 mol/L ODPA-modified zeolite 5A (dark orange line) after modification without THF washing.

Figure S2. TG, DSC, and DTG curves of modified zeolite 5A materials with different concentrations and without THF washing after modification process: (A) modified zeolite 5A with 0.01 mol/L of ODPA, (B) 0.005 mol/L ODPA-modified zeolite 5A, and (C) 0.001 mol/L ODPA-modified zeolite 5A.

Figure S3. EDS spectra and elemental compositions of pristine and ODPA-modified zeolite 5A materials THF washed: (A) pristine zeolite 5A, (B) 0.01 mol/L ODPA-modified zeolite 5A, (C) 0.005 mol/L ODPA-modified zeolite 5A, and (D) 0.001 mol/L ODPA-modified zeolite 5A.

Figure S4. EDS spectra and elemental compositions of modified zeolite 5A without THF washing after modification process: (A) 0.01 mol/L ODPA-modified zeolite 5A, (B) 0.005 mol/L ODPA-modified zeolite 5A, and (C) 0.001 mol/L ODPA-modified zeolite 5A.

Figure S5. Element distribution of pristine and modified zeolite 5A with THF washing after the modification process: (A) pristine zeolite 5A, (B) 0.01 mol/L ODPA-modified zeolite 5A, (C) 0.005 mol/L ODPA-modified zeolite 5A, and (D) 0.001 mol/L ODPA-modified zeolite 5A.

Figure S6. Element distribution of modified zeolite 5A without THF washing after modification process: (A) 0.01 mol/L ODPA-modified zeolite 5A, (B) 0.005 mol/L ODPA-modified zeolite 5A, and (C) 0.001 mol/L ODPA-modified zeolite 5A.

Figure S7. Adsorption isotherms of (A) CO_2 and (B) C_3H_6 in pristine and the ODPA-modified zeolite 5A material at 298 K with different ODPA concentrations without washing after modification.

Figure S8. adsorption isotherms of (A) CO_2 and (B) C_3H_6 in pristine and the ODPA-modified zeolite 5A material measured at 323 K; Impact of ODPA concentration and washing Procedure. The materials washed with tetrahydrofuran after modification are marked with an asterisk (*).

Figure S9. adsorption isotherms of (A) CO_2 and (B) C_3H_6 in pristine and the ODPA-modified zeolite 5A material measured at 348 K; Impact of ODPA concentration and washing Procedure. The materials washed with tetrahydrofuran after modification are marked with an asterisk (*).

Figure S10. adsorption isotherms of (A) CO_2 and (B) C_3H_6 in pristine and the ODPA-modified zeolite 5A material measured at 373 K; Impact of ODPA concentration and washing Procedure. The materials washed with tetrahydrofuran after modification are marked with an asterisk (*).

Figure S11. CO_2 adsorption data fitted with the sips isotherm model across all temperatures: (A) 298 K, (B) 323 K, (C) 348 K, and (D) 373 K, reflecting the heterogeneous nature of adsorption sites in pristine and the ODPA-modified zeolite 5A material, and with asterisks (*) indicating THF-washed materials.

Figure S12. C_3H_6 adsorption data fitted with the sips isotherm model across all temperatures: (A) 298 K, (B) 323 K, (C) 348 K, and (D) 373 K, reflecting the heterogeneous nature of adsorption sites in pristine and the ODPA-modified zeolite 5A material, and with asterisks (*) indicating THF-washed materials.

Figure S13. CO_2 adsorption data fitted with the langmuir isotherm model across all temperatures: (A) 298 K, (B) 323 K, (C) 348 K, and (D) 373 K. Asterisks (*) indicating THF-washed materials.

Figure S14. C_3H_6 adsorption data fitted with the langmuir isotherm model across all temperatures: (A) 298 K, (B) 323 K, (C) 348 K, and (D) 373 K. Asterisks (*) indicating THF-washed materials.

Figure S15. Van't Hoff plots of ln(k) versus $1/T$ for (A) CO_2 and (B) C_3H_6 adsorption in pristine and the ODPA-modified zeolite 5A material without THF washing after modification.

Figure S16. Isosteric adsorption heat of (A) CO_2 and (B) C_3H_6 in pristine and the ODPA-modified zeolite 5A material without THF washing after modification.

Figure S17. Separation factor (R_L) vs. initial concentration (C_0) for adsorption in pristine and the ODPA-modified zeolite 5A material at 298 K: (A) CO_2 and (B) C_3H_6. The asterisks (*) indicating THF-washed materials. The adsorption rate constant from Sips model was used for the calculation of separation factor.

Figure S18. Time-dependent uptake of (A) CO_2 and (B) C_3H_6 in pristine and the ODPA-modified zeolite 5A material at 298 K with different ODPA concentrations without washing after modification.

Figure S19. Time-dependent uptake of (A) CO_2 and (B) C_3H_6 in pristine and the ODPA-modified zeolite 5A material at 323 K and 100 kPa.

Figure S20. Time-dependent uptake of (A) CO_2 and (B) C_3H_6 in pristine and the ODPA-modified zeolite 5A material at 348 K and 100 kPa.

Figure S21. Time-dependent uptake of (A) CO_2 and (B) C_3H_6 in pristine and the ODPA-modified zeolite 5A material at 373 K and 100 kPa.

Figure S22. Time-dependent C_3H_6 uptake in pristine zeolite 5A, 0.005 mol/L ODPA-modified zeolite 5A (THF-washed), and 0.01 mol/L ODPA-modified zeolite 5A (THF-washed), based on zeolite 5A mass; showing unchanged equilibrium uptake and reduced adsorption rate after ODPA modification.

Figure S23. Kinetic selectivity of CO_2/C_3H_6 adsorption in pristine and the ODPA-modified zeolite 5A material at varying temperatures: (A) 323 K, (B) 348 K, (C), and 373 K; THF-washed materials are marked by an asterisk (*).

Figure S24. Linear fitting of ln(q_e - q_t) versus time for the adsorption uptake in pristine and ODPA-modified zeolite 5A without THF washing after modification at 298 K using the PFO model: (A) CO_2 and (B) C_3H_6.

Figure S25. Linear fitting of t/q_t versus time for the adsorption uptake in pristine and ODPA-modified zeolite 5A without THF washing after modification at 298 K using the PSO model: (A) CO_2 and (B) C_3H_6.

Figure S26. Intraparticle diffusion model plots for (A) CO_2 and (B) C_3H_6 adsorption in pristine and ODPA-modified zeolite 5A without THF washing after modification at 298 K. The asterisks (*) indicate the THF-washed materials.

Figure S27. Arrhenius plots of ln(*k*) versus 1/*T* for PFO kinetic model. (A) CO_2 and (B) C_3H_6 adsorption in pristine and the ODPA-modified zeolite 5A material (washed with THF). Asterisks (*) indicate THF-washed materials.

Figure S28. Arrhenius plots of ln(*k*) versus 1/*T* for the PSO kinetic model. (A) CO_2 and (B) C_3H_6 adsorption in pristine and the ODPA-modified zeolite 5A material without THF washing after modification.

Figure S29. Arrhenius plots of ln(*k*) versus 1/*T* for the PFO kinetic model. (A) CO_2 and (B) C_3H_6 adsorption in pristine and the ODPA-modified zeolite 5A material without THF washing after modification.

Supporting Tables

Table S1 Thermal Analysis Data for pristine and ODPA-Modified Zeolite 5A; (*) denotes THF-washed materials.

Table S2 Element composition (at.%) of pristine and the ODPA-modified zeolite 5A material from XPS analysis.

Table S3 Adsorption isotherms parameters of the Sips model for CO_2 adsorption in pristine and modified 5A zeolite. (The asterisk (*) denotes THF-washed materials.)

Table S4 Adsorption isotherms parameters of the Sips model for C_3H_6 adsorption in pristine and modified 5A zeolite. (The asterisk (*) denotes THF-washed materials.)

Table S5 Adsorption isotherms parameters of the Langmuir model for CO_2 adsorption in pristine and modified 5A zeolite. (The asterisk (*) denotes THF-washed materials.)

Table S6 Adsorption isotherms parameters of the Langmuir model for C_3H_6 adsorption in pristine and modified 5A zeolite. (The asterisk (*) denotes THF-washed materials.)

Table S7. Thermodynamic parameters for C_3H_6 adsorption in pristine and the ODPA-modified zeolite 5A material. (The asterisk (*) denotes THF-washed materials.)

Table S8 The diffusion parameters for CO_2 adsorption in pristine and the ODPA-modified zeolite 5A material at 298 K. The asterisks (*) indicate the THF-washed materials.

Table S9 The diffusion parameters for C_3H_6 adsorption in pristine and the ODPA-modified zeolite 5A material at 298 K. The asterisks (*) indicate the THF-washed materials.

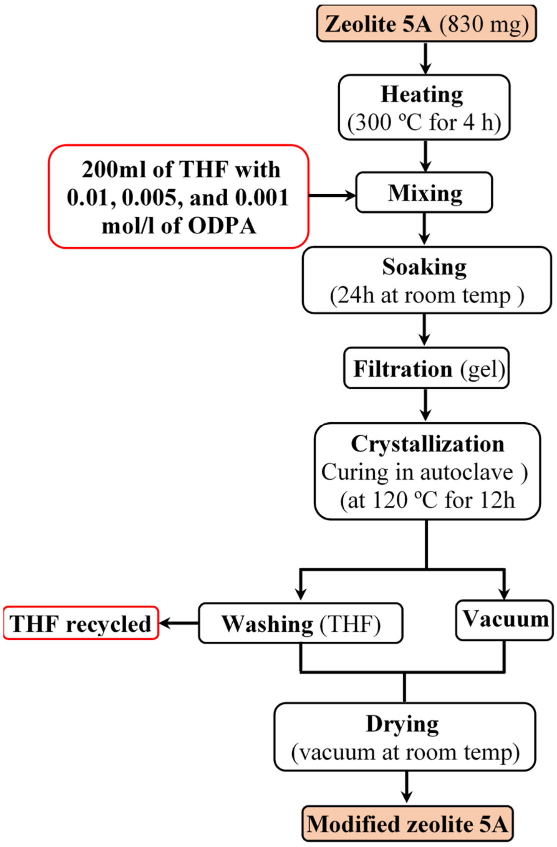

Scheme S1. Schematic flow diagram depicting the surface modification of zeolite 5A with *n*-octadecylphosphonic acid.

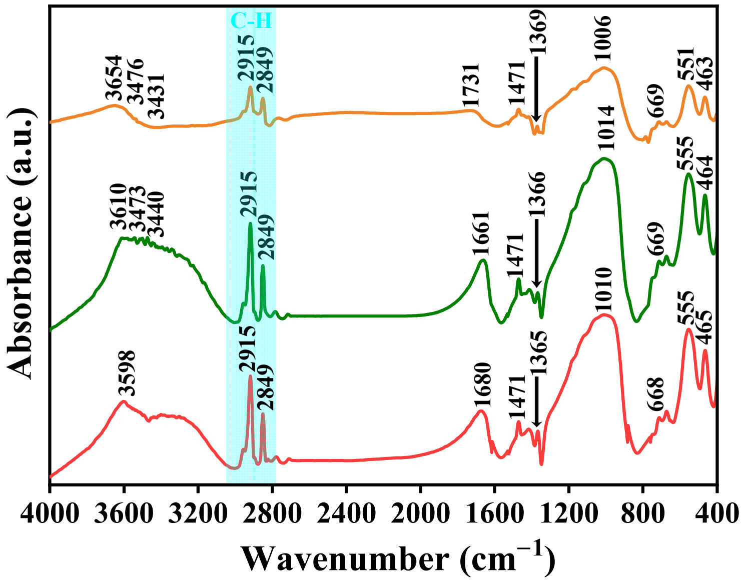

Figure S1. FTIR spectra of 0.01 mol/L ODPA-modified zeolite 5A (red line), 0.005 mol/L ODPA-modified zeolite 5A (olive line), and 0.001 mol/L (dark orange line) after modification without THF washing. The spectra display shifts in vibrational bands, indicating ODPA incorporation into the zeolite framework. The concentration-dependent variations in band intensity and position suggest different levels of interaction between ODPA and the zeolite.

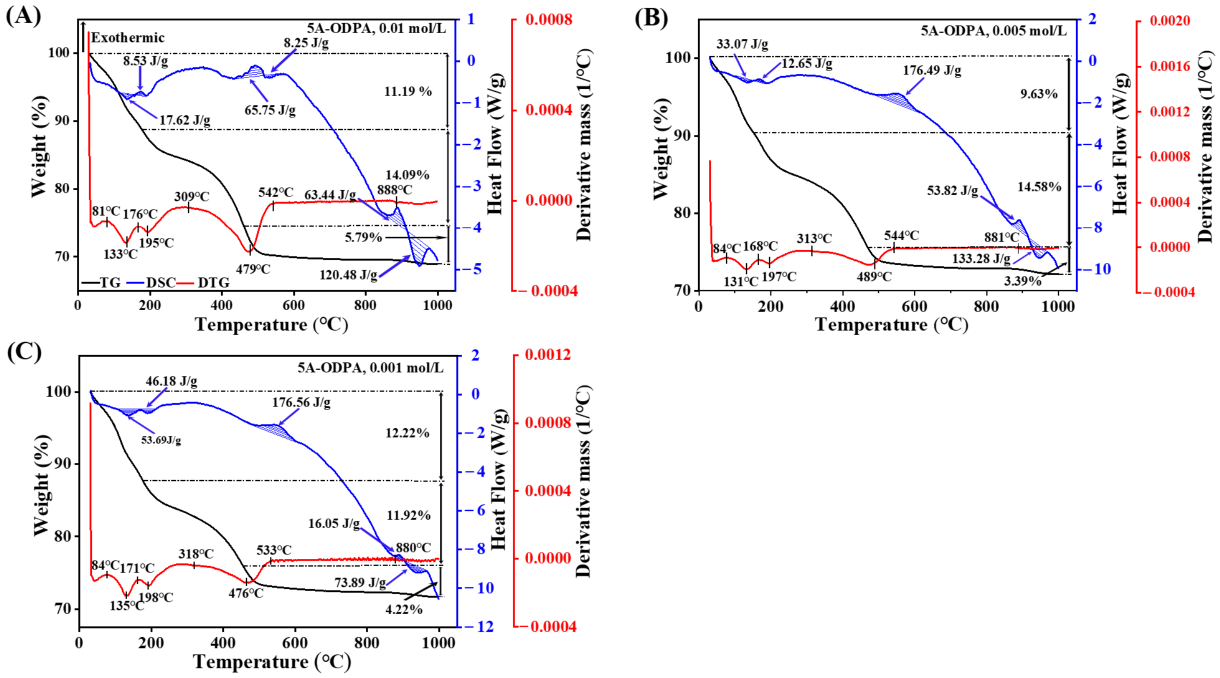

Figure S2. TG, DSC, and DTG curves of modified zeolite 5A materials with different concentrations and without THF washing after modification process: (A) modified zeolite 5A with 0.01 mol/L of ODPA, (B) 0.005 mol/L ODPA-modified zeolite 5A, and (C) 0.001 mol/L ODPA-modified zeolite 5A. The DSC curve shows distinct enthalpy changes for each material, indicating the thermal behavior differences due to ODPA concentration without washing process.

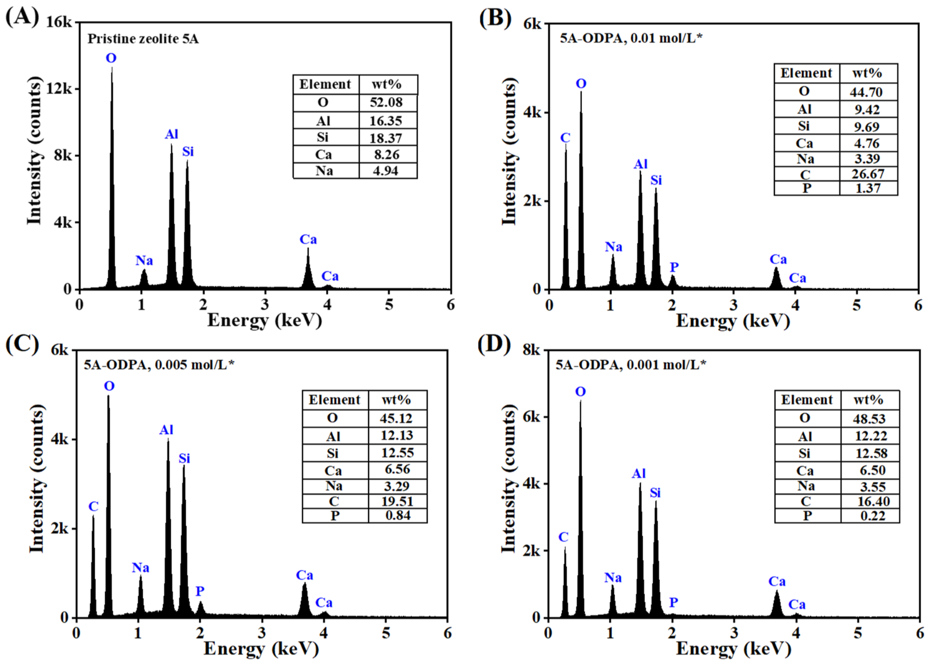

Figure S3. EDS spectra and elemental compositions of pristine and THF-washed modified materials: (A) pristine zeolite 5A, (B) 0.01 mol/L ODPA-modified zeolite 5A, (C) 0.005 mol/L ODPA-modified zeolite 5A, and (D) 0.001 mol/L ODPA-modified zeolite 5A.

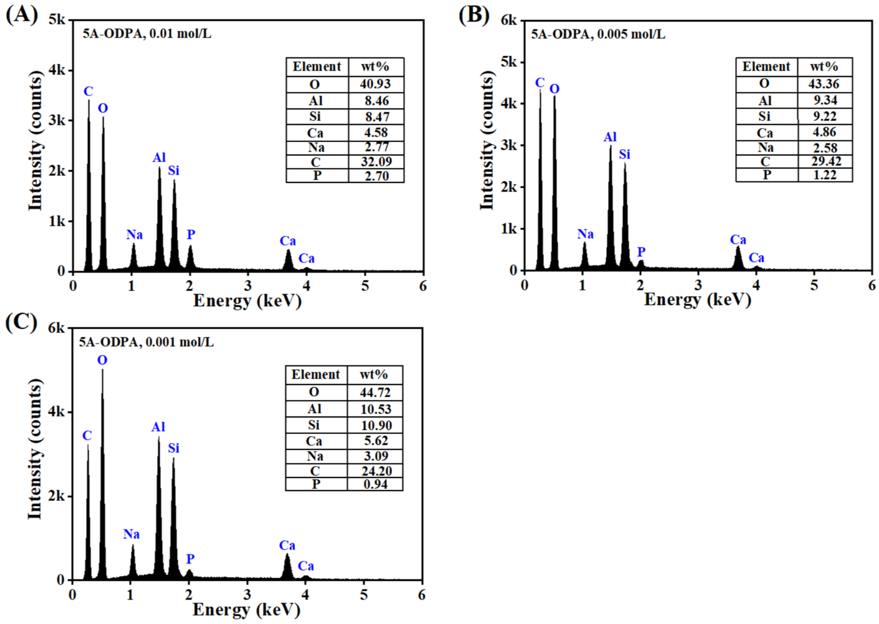

Figure S4. EDS spectra and elemental compositions of modified zeolite 5A without THF washing after modification process: (A) 0.01 mol/L ODPA-modified zeolite 5A, (B) 0.005 mol/L ODPA-modified zeolite 5A, and (C) 0.001 mol/L ODPA-modified zeolite 5A.

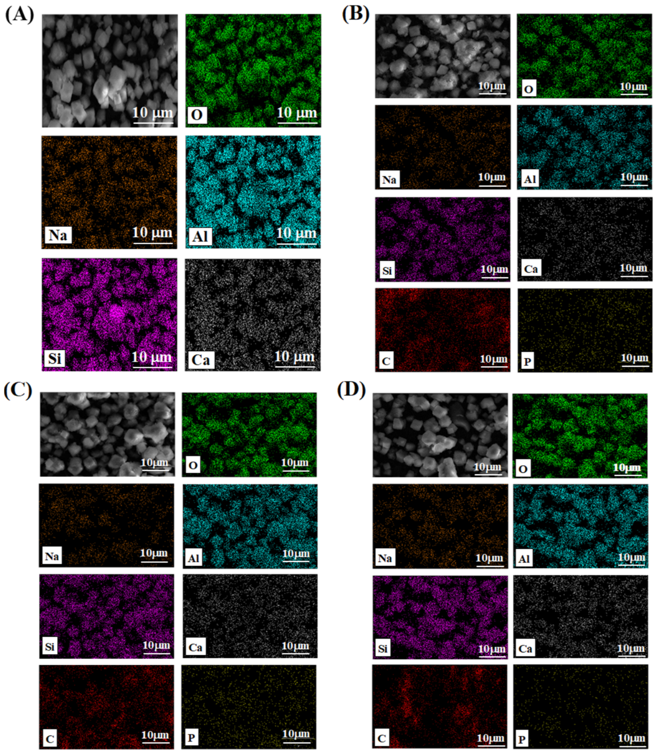

Figure S5. Element distribution of pristine and modified zeolite 5A with THF washing after the modification process: (A) pristine zeolite 5A, (B) 0.01 mol/L ODPA-modified zeolite 5A, (C) 0.005 mol/L ODPA-modified zeolite 5A, and (D) 0.001 mol/L ODPA-modified zeolite 5A. X-ray signals are collected to create a detailed map showing where specific elements are located within the material. This is especially useful for studying compositional variations in materials.

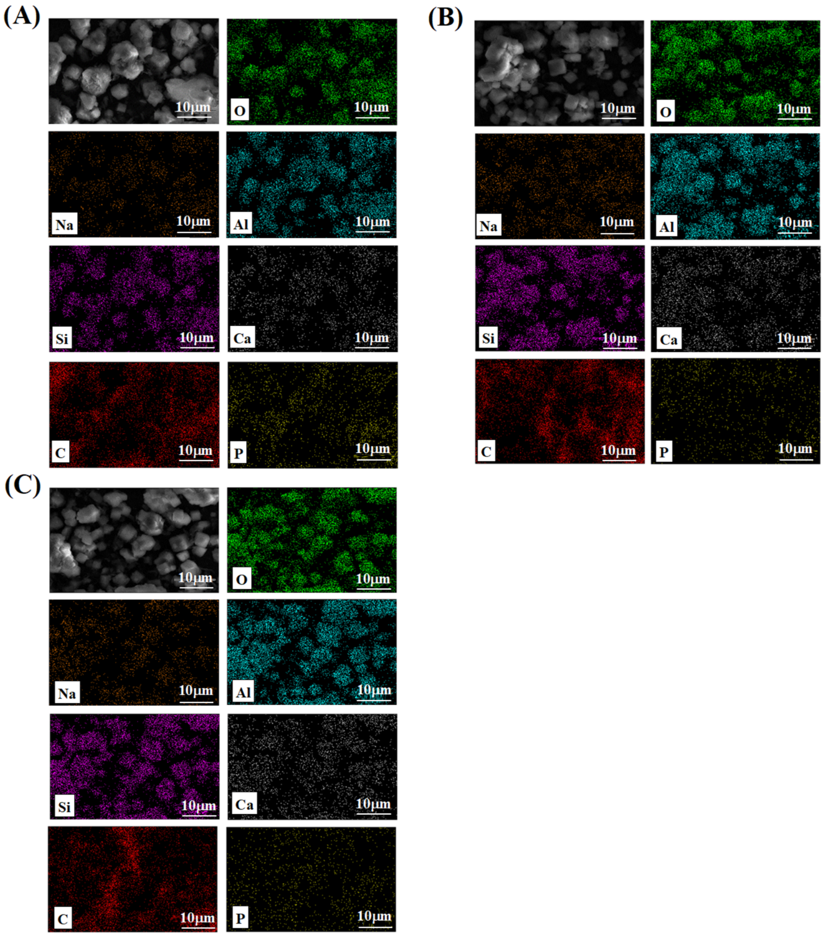

Figure S6. Element distribution of modified zeolite 5A without THF washing after modification process: (A) 0.01 mol/L ODPA-modified zeolite 5A, (B) 0.005 mol/L ODPA-modified zeolite 5A, and (C) 0.001 mol/L ODPA-modified zeolite 5A.

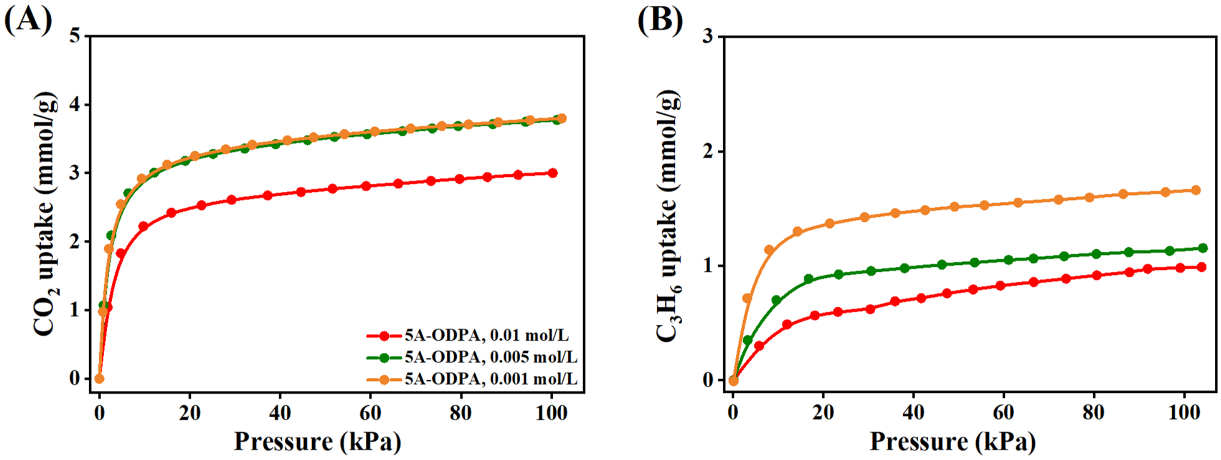

Figure S7. Adsorption isotherms of (A) CO_2 and (B) C_3H_6 in pristine and the ODPA-modified zeolite 5A material at 298 K with different ODPA concentrations without washing after modification.

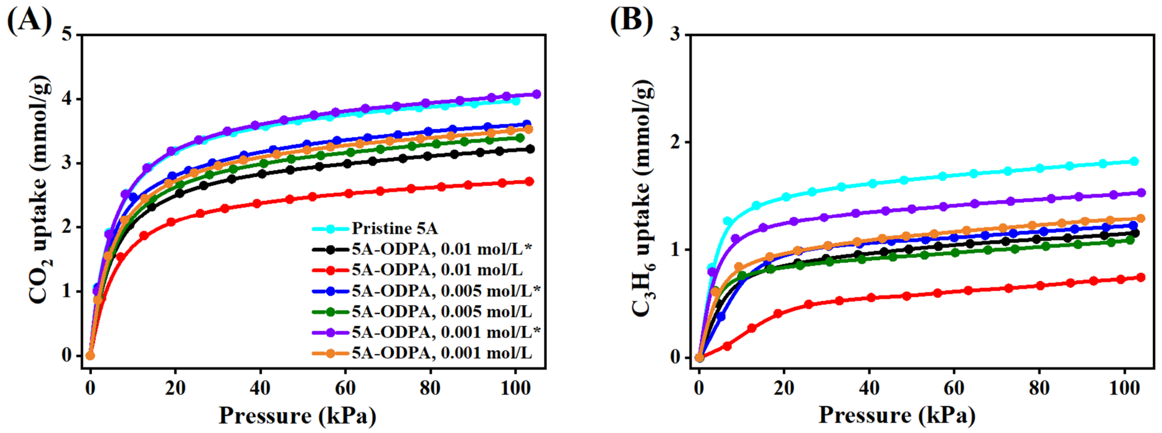

Figure S8. adsorption isotherms of (A) CO_2 and (B) C_3H_6 in pristine and the ODPA-modified zeolite 5A material measured at 323 K; Impact of ODPA concentration and washing Procedure. The materials washed with tetrahydrofuran after modification are marked with an asterisk (*).

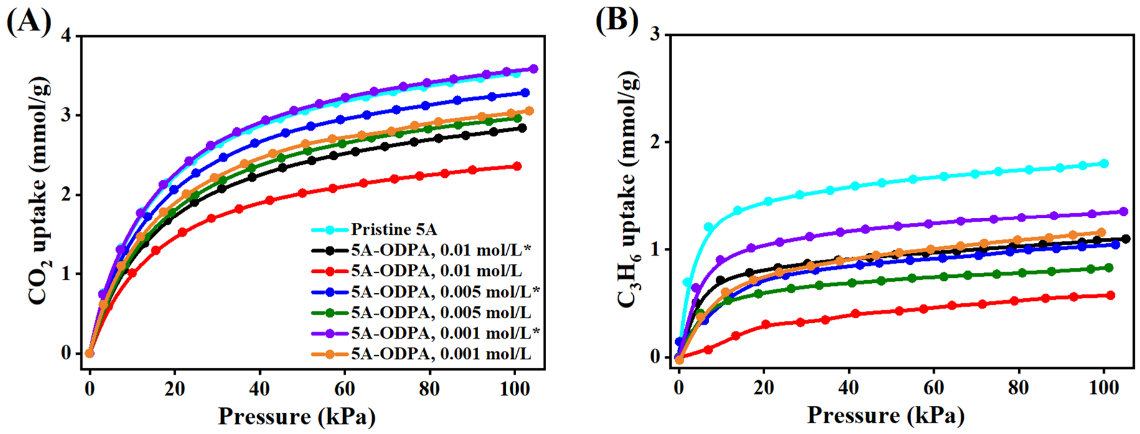

Figure S9. adsorption isotherms of (A) CO_2 and (B) C_3H_6 in pristine and the ODPA-modified zeolite 5A material measured at 348 K; Impact of ODPA concentration and washing Procedure. The materials washed with tetrahydrofuran after modification are marked with an asterisk (*).

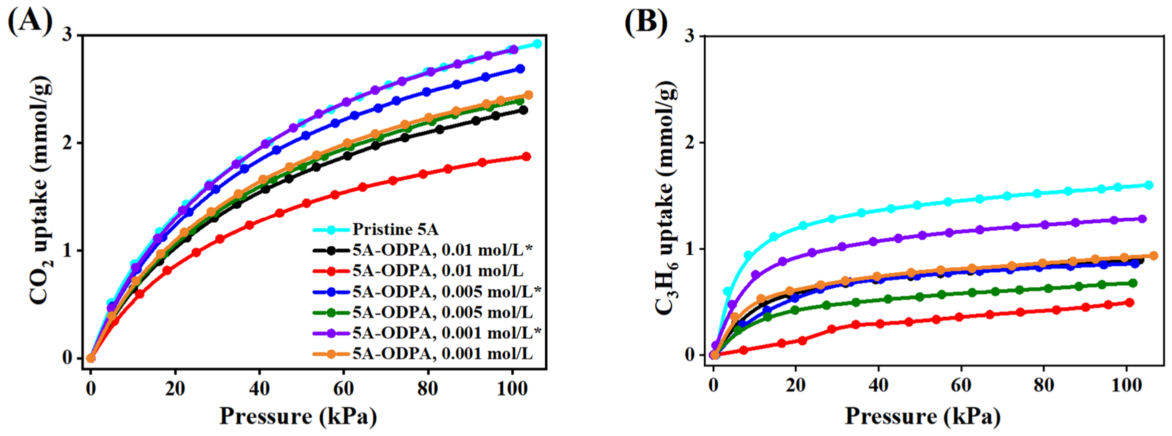

Figure S10. adsorption isotherms of (A) CO_2 and (B) C_3H_6 in pristine and the ODPA-modified zeolite 5A material measured at 373 K; Impact of ODPA concentration and washing Procedure. The materials washed with tetrahydrofuran after modification are marked with an asterisk (*).

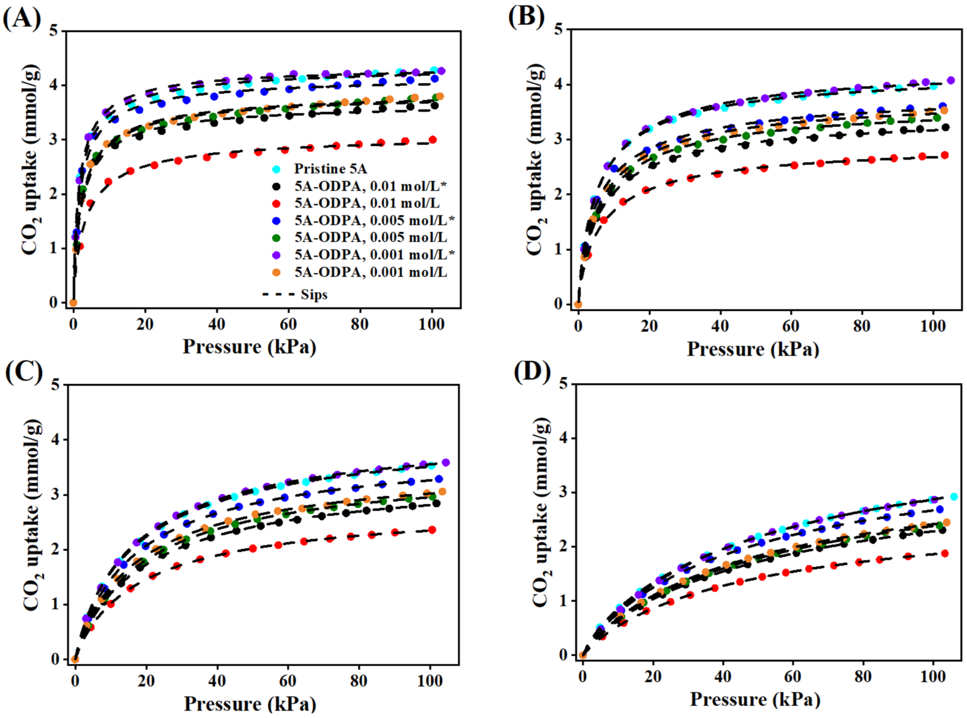

Figure S11. CO_2 adsorption data fitted with the Sips isotherm model across all temperatures: (A) 298 K, (B) 323 K, (C) 348 K, and (D) 373 K, reflecting the heterogeneous nature of adsorption sites in pristine and the ODPA-modified zeolite 5A material, and with asterisks (*) indicating THF-washed materials.

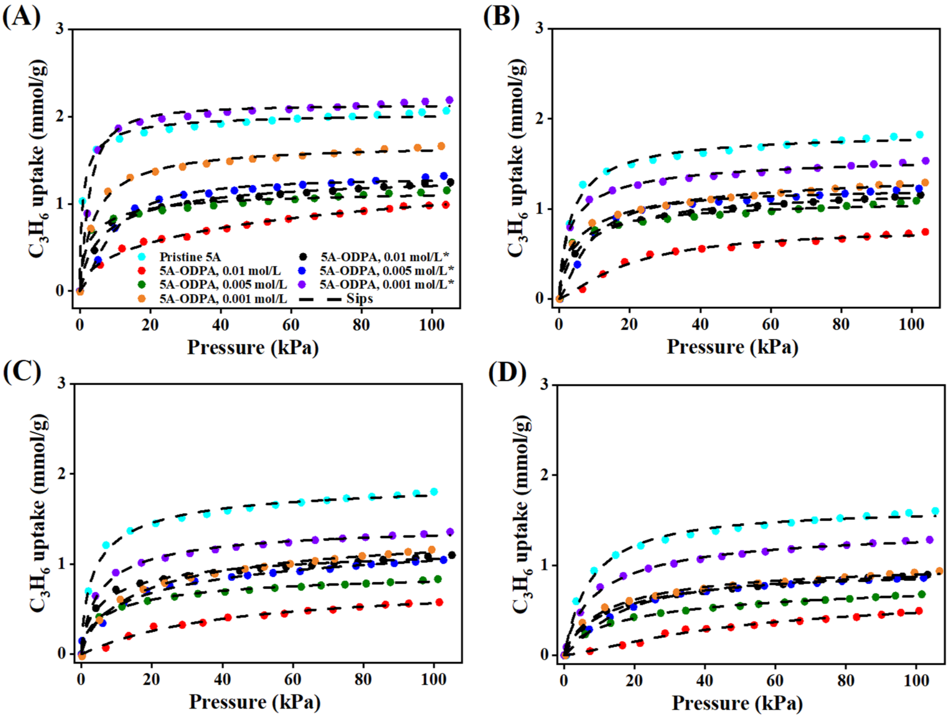

Figure S12. C_3H_6 adsorption data fitted with the Sips isotherm model across all temperatures: (A) 298 K, (B) 323 K, (C) 348 K, and (D) 373 K, reflecting the heterogeneous nature of adsorption sites in pristine and the ODPA-modified zeolite 5A material, and with asterisks (*) indicating THF-washed materials.

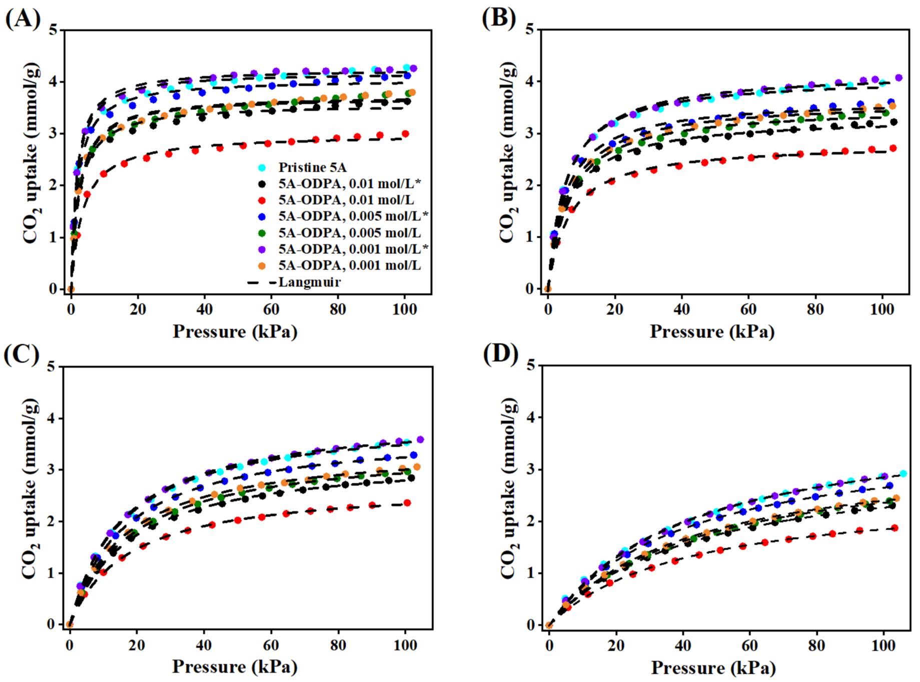

Figure S13. CO_2 adsorption data fitted with the Langmuir isotherm model across all temperatures: (A) 298 K, (B) 323 K, (C) 348 K, and (D) 373 K, showing good agreement at higher temperatures (348 and 373 K), indicating monolayer adsorption on homogeneous sites of pristine and the ODPA-modified zeolite 5A material, and with asterisks (*) indicating THF-washed materials.

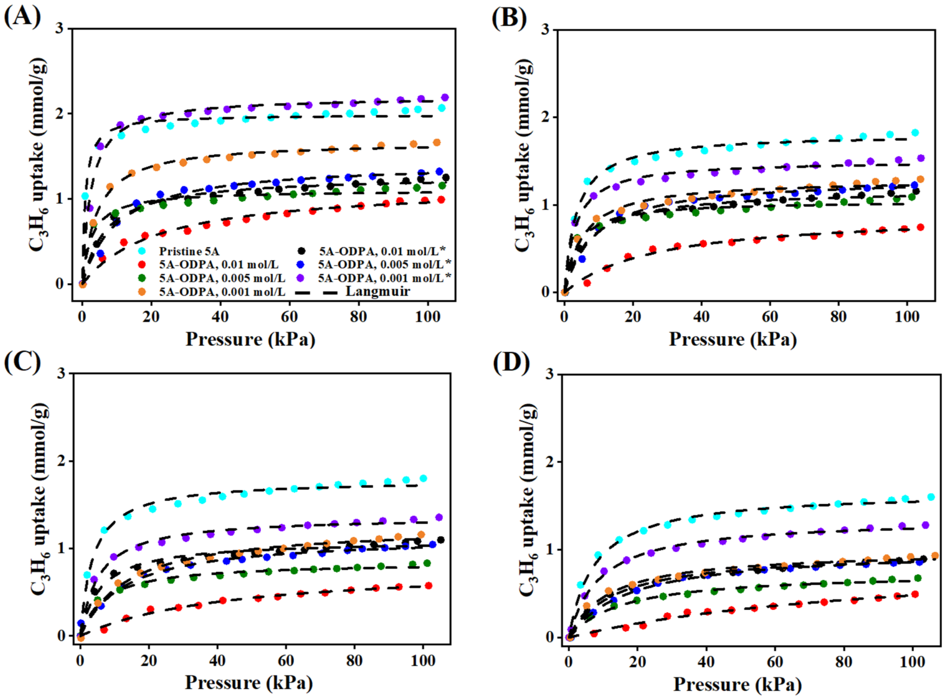

Figure S14. C_3H_6 adsorption data fitted with the Langmuir isotherm model across all temperatures: (A) 298 K, (B) 323 K, (C) 348 K, and (D) 373 K, showing good agreement at higher temperatures (348 and 373 K), indicating monolayer adsorption on homogeneous sites of pristine and the ODPA-modified zeolite 5A material, and with asterisks (*) indicating THF-washed materials.

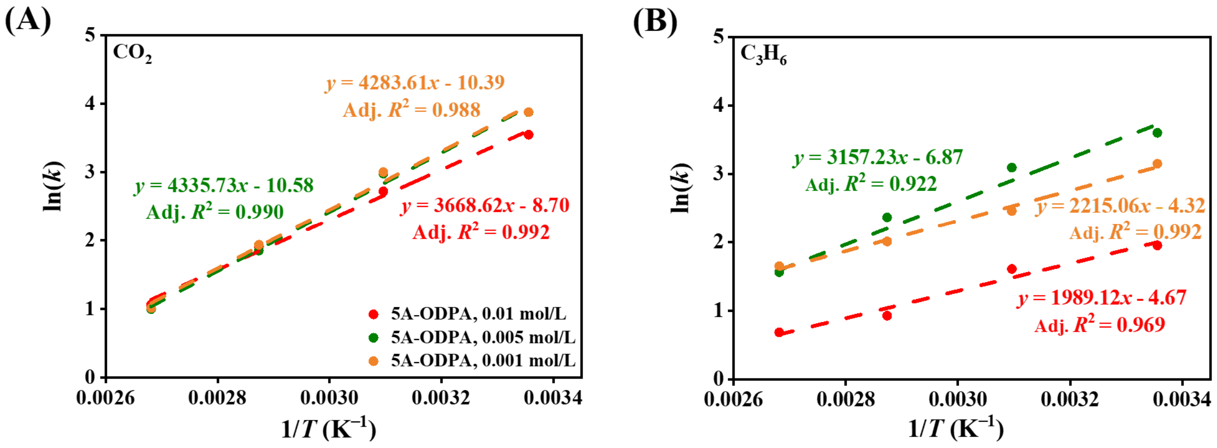

Figure S15. Van't Hoff plots of ln(k) versus 1/T for (A) CO_2 and (B) C_3H_6 adsorption in pristine and the ODPA-modified zeolite 5A material without THF washing after modification.

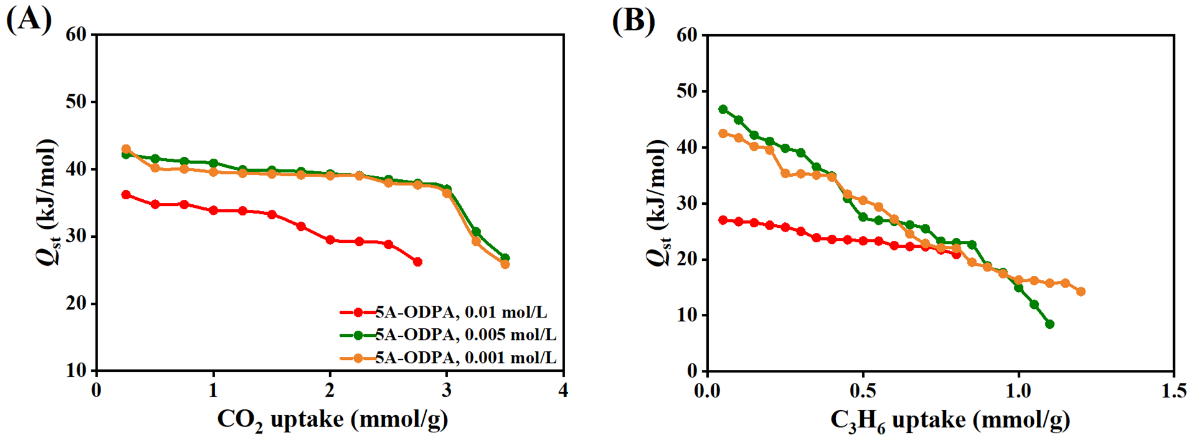

Figure S16. Isosteric adsorption heat vs. uptake for adsorption of (A) CO_2 and (B) C_3H_6 in pristine and the ODPA-modified zeolite 5A materials without THF washing after modification.

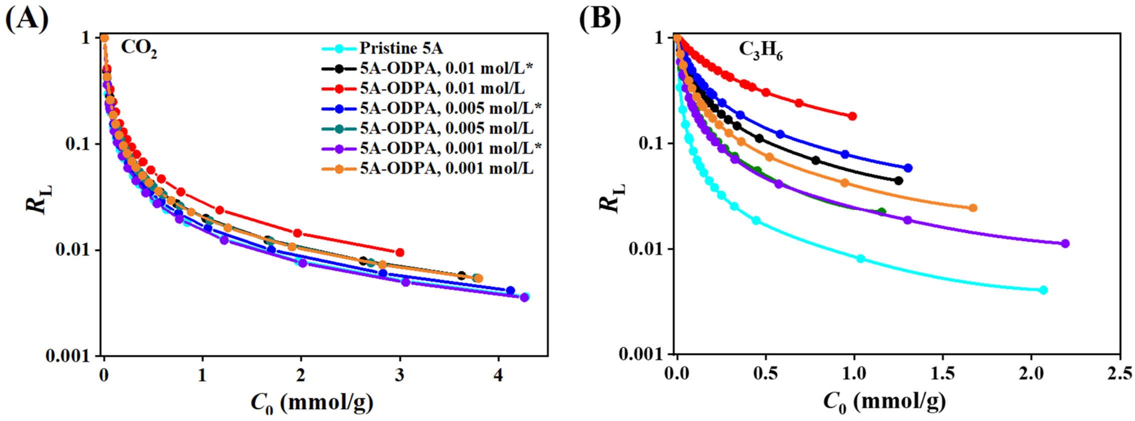

Figure S17. Separation factor (R_L) vs. initial concentration (C_0) for adsorption in pristine and the ODPA-modified zeolite 5A material at 298 K: (A) CO_2 and (B) C_3H_6. The asterisks (*) indicating THF-washed materials. The adsorption rate constant from Sips model was used for the calculation of separation factor.

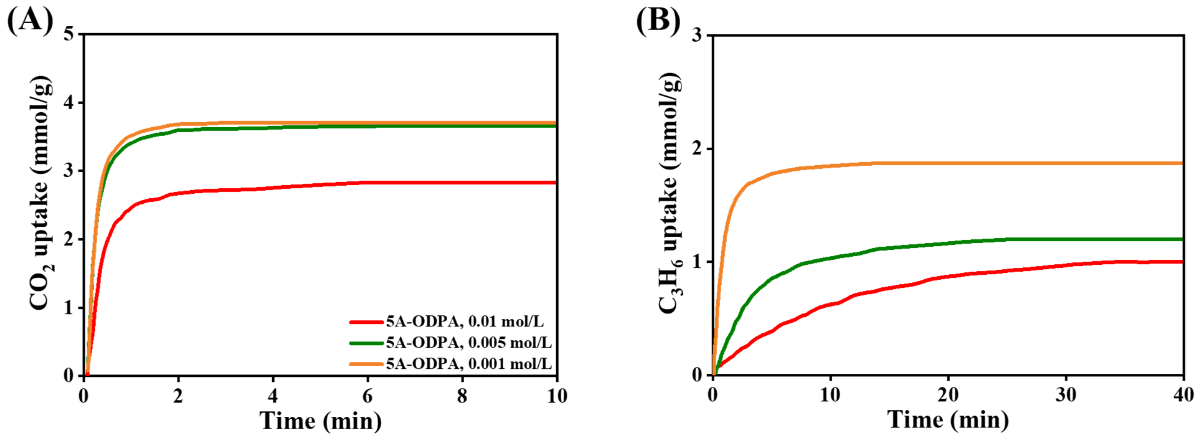

Figure S18. Time-dependent uptake of (A) CO_2 and (B) C_3H_6 in pristine and the ODPA-modified zeolite 5A material at 298 K with different ODPA concentrations without washing after modification.

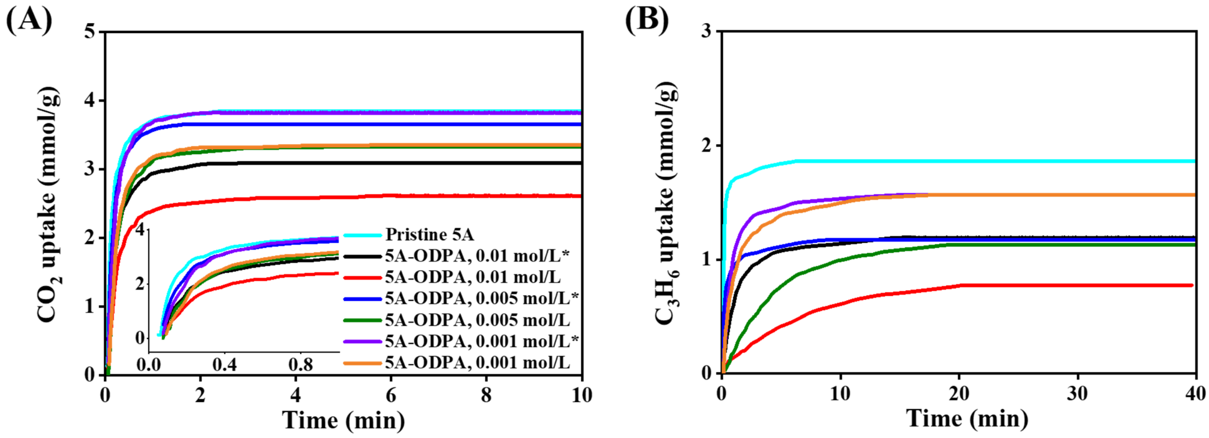

Figure S19. Time-dependent uptake of (A) CO_2 and (B) C_3H_6 in pristine and the ODPA-modified zeolite 5A material at 323 K and 100 kPa.

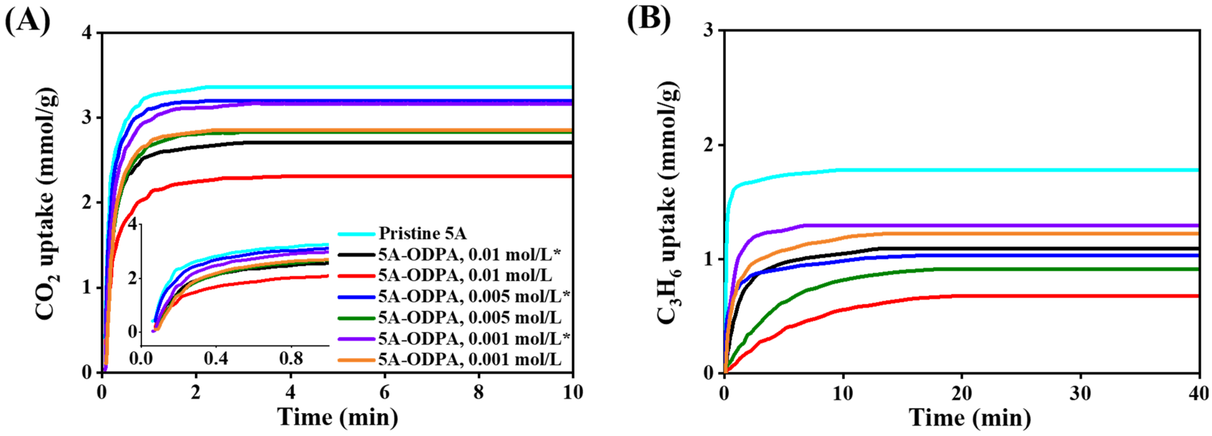

Figure S20. Time-dependent uptake of (A) CO_2 and (B) C_3H_6 in pristine and the ODPA-modified zeolite 5A material at 348 K and 100 kPa.

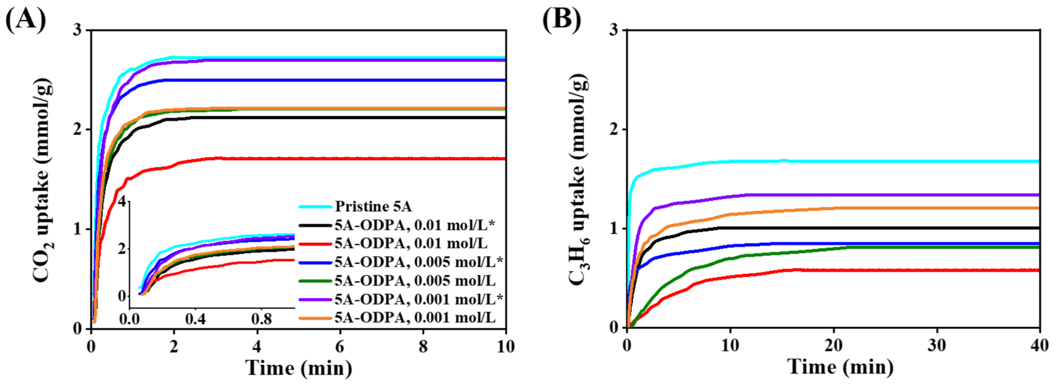

Figure S21. Time-dependent uptake of (A) CO_2 and (B) C_3H_6 in pristine and the ODPA-modified zeolite 5A material at 373 K and 100 kPa.

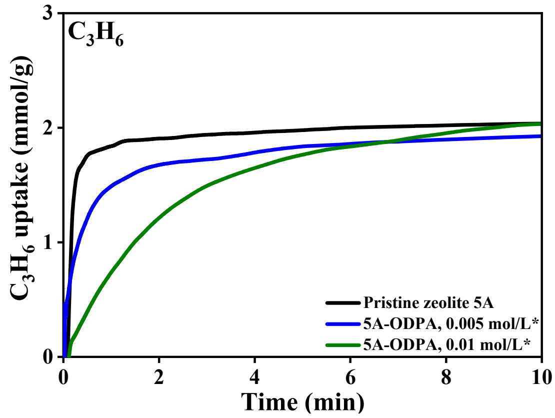

Figure S22. Time-dependent C_3H_6 uptake in pristine zeolite 5A, 0.005 mol/L ODPA-modified zeolite 5A (THF-washed), and 0.01 mol/L ODPA-modified zeolite 5A (THF-washed), based on zeolite 5A mass; showing unchanged equilibrium uptake and reduced adsorption rate after ODPA modification.

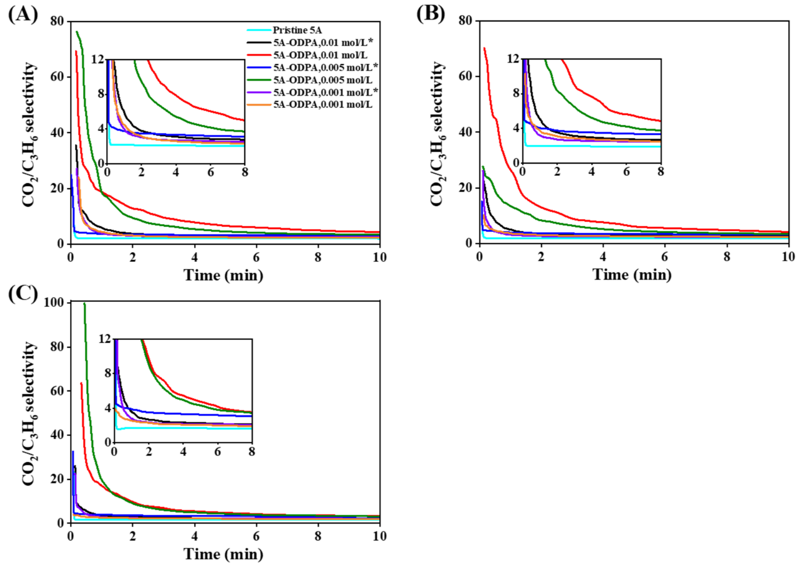

Figure S23. Ideal selectivity of CO_2/C_3H_6 adsorption in pristine and the ODPA-modified zeolite 5A material at varying temperatures: (A) 323 K, (B) 348 K, (C), and 373 K; THF-washed materials are marked by an asterisk (*).

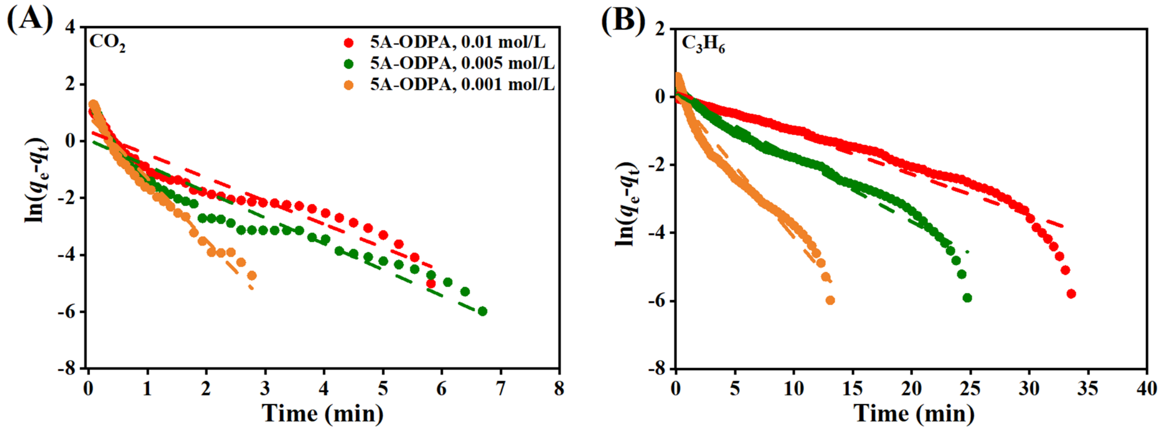

Figure S24. Linear fitting of ln(q_e - q_t) versus time for the adsorption uptake in pristine and ODPA-modified zeolite 5A zeolite 5A without THF washing after modification at 298 K using the PFO model: (A) CO_2 and (B) C_3H_6.

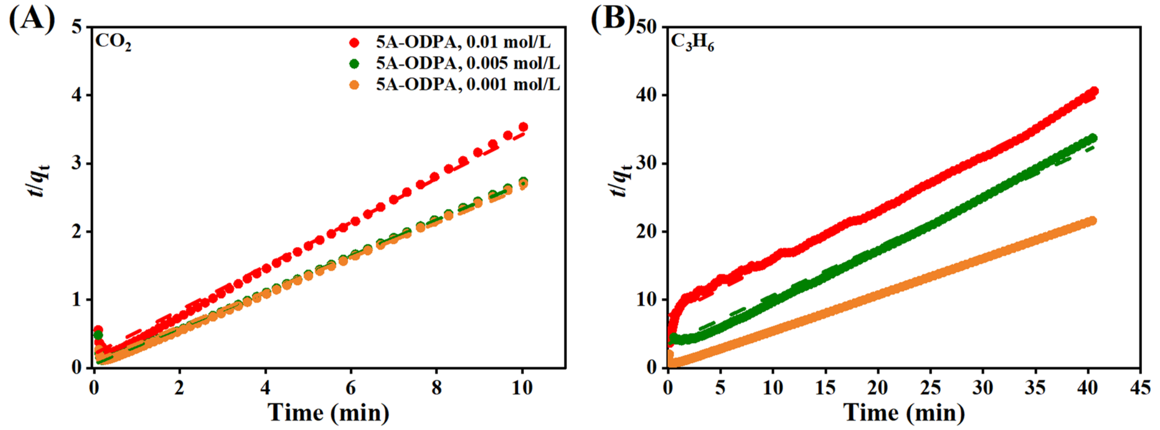

Figure S25. Linear fitting of t/q_t versus time for the adsorption uptake in pristine and ODPA-modified zeolite 5A zeolite 5A without THF washing after modification at 298 K using the PSO model: (A) CO_2 and (B) C_3H_6.

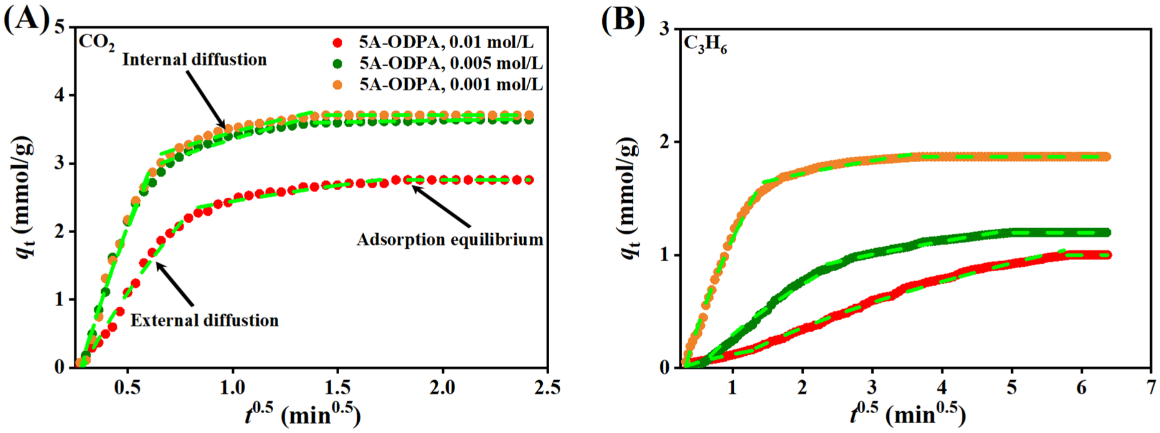

Figure S26. Intraparticle diffusion model plots for (A) CO_2 and (B) C_3H_6 adsorption in pristine and ODPA-modified zeolite 5A zeolite 5A without THF washing after modification at 298 K. The asterisks (*) indicate the THF-washed materials.

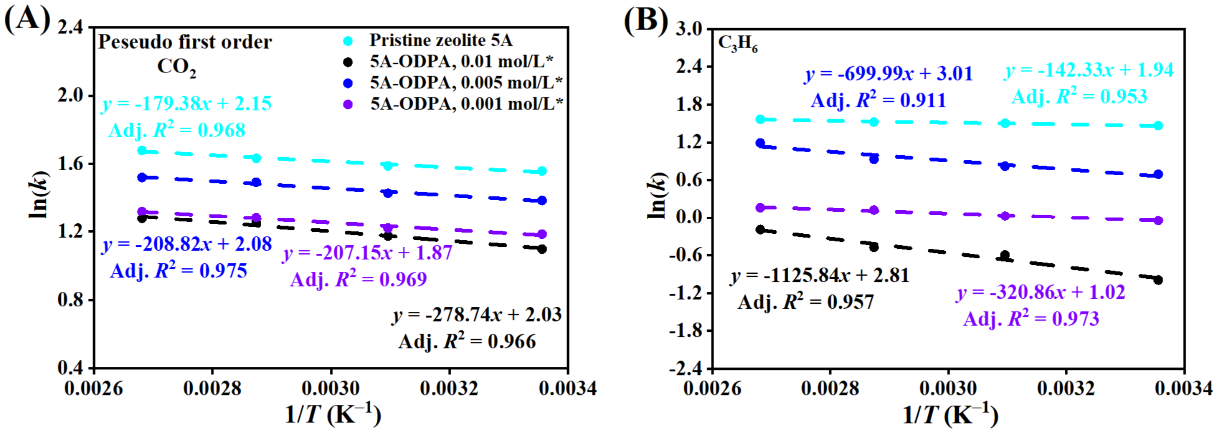

Figure S27. Arrhenius plots of ln(k) versus 1/T for PFO kinetic model. (A) CO_2 and (B) C_3H_6 adsorption in pristine and the ODPA-modified zeolite 5A material (washed with THF). Asterisks (*) indicate THF-washed materials.

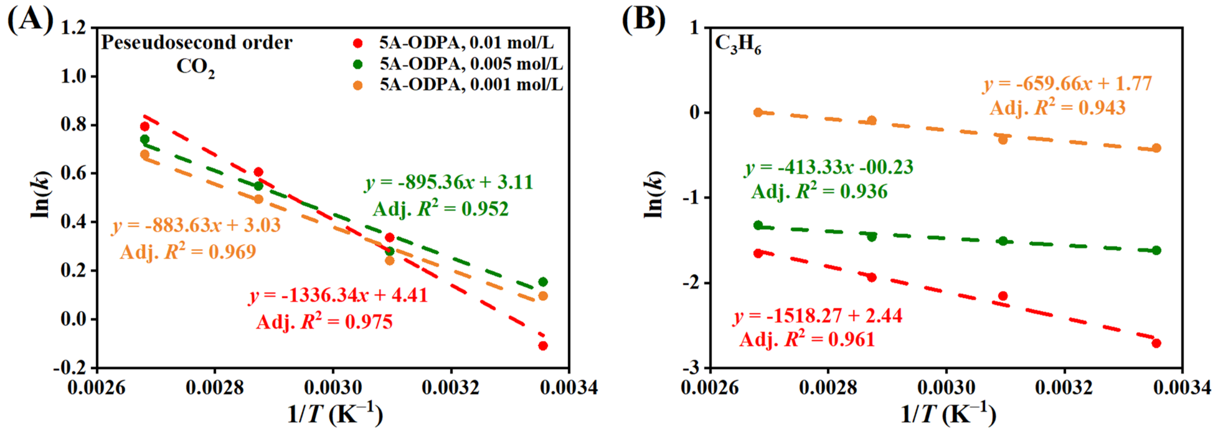

Figure S28. Arrhenius plots of ln(k) versus 1/T for the PSO kinetic model. (A) CO_2 and (B) C_3H_6 adsorption in pristine and the ODPA-modified zeolite 5A material without THF washing after modification.

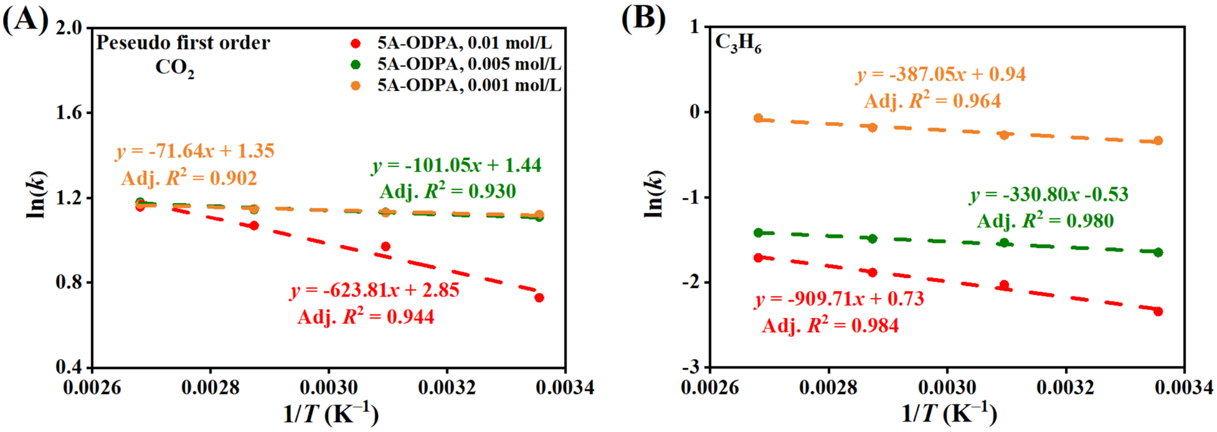

Figure S29. Arrhenius plots of ln(k) versus 1/T for the PFO kinetic model. (A) CO_2 and (B) C_3H_6 adsorption in pristine and the ODPA-modified zeolite 5A material without THF washing after modification.

Table S1 Thermal Analysis Data for pristine and ODPA-modified zeolite 5A; (*) denotes THF-washed materials.

Material	Mass Loss (%)			DSC Peaks (Temp. (℃), Enthalpy (J/g), Assignment)
	Region I	Region II	Region III	
**Pristine zeolite 5A**	15.43	2.77		150 and 200 ℃: H_2O removal; 900 and 950 ℃: zeolite crystal transition
**5A-ODPA, 0.01 mol/L**	11.19	14.09	5.79	140 ℃: H_2O removal; 500 ℃: ODPA transition; 900 and 950 ℃: zeolite crystal transition
**5A-ODPA, 0.01 mol/L***	11.91	18.71	4.37	138 and 215 ℃: H_2O removal; 450-650 ℃: ODPA transition
**5A-ODPA, 0.005 mol/L**	9.63	14.58	3.39	130 ℃: H_2O removal; 550 ℃: ODPA transition; 900 and 950 ℃: zeolite crystal transition
**5A-ODPA, 0.005 mol/L***	10.98	10.55	3.91	135 and 200 ℃: H_2O removal; 450 ℃: ODPA transitions); 900 and 950 ℃: zeolite crystal transition
**5A-ODPA, 0.001 mol/L**	12.22	11.92	4.22	135 and 185 ℃: H_2O removal; 550 ℃: ODPA transitions; 900 and 950 ℃: zeolite crystal transition
**5A-ODPA, 0.001 mol/L***	7.87	6.68	3.95	150 ℃: H_2O removal; 450 and 750 ℃: ODPA transition; 850 and 900 ℃: zeolite crystal transition

Table S2 Element composition (at.%) of pristine and the ODPA-modified unwashed zeolite 5A material from XPS analysis.

Material	at%						
	C	O	Si	Al	Na	Ca	P
**Pristine zeolite 5A**	22.17	46.57	12.59	13.74	2.08	2.85	0.00
**5A-ODPA, 0.01 mol/L**	67.78	18.60	3.70	4.51	N.D.	1.94	3.70
**5A-ODPA, 0.005 mol/L**	63.54	21.47	5.16	4.93	N.D.	1.90	3.01
**5A-ODPA, 0.001 mol/L**	58.05	25.14	5.91	5.39	0.99	1.68	2.84

The abbreviation N.D. refers to Not Detected.

Table S3 Adsorption isotherms parameters of the Sips model for CO_2 adsorption in pristine and modified 5A zeolite. (The asterisk (*) denotes THF-washed materials.)

Material	Temperature (K)	Adj. R^2	ΔQ	Model parameters		
				q_m (mmol/g)	k_S (kPa^{-1})	n
**pristine zeolite 5A**	298	0.9971	3.94×10^{-3}	4.4001	0.6415	0.7566
	323	0.9996	5.71×10^{-4}	4.2716	0.2265	0.8546
	348	0.9999	8.06×10^{-5}	4.2674	0.0759	0.8939
	373	0.9999	4.90×10^{-5}	4.8731	0.0316	0.8296
**5A-ODPA, 0.01 mol/L**	298	0.9958	6.66×10^{-3}	3.1185	0.3484	0.8286
	323	0.9991	4.79×10^{-4}	2.9766	0.2022	0.8208
	348	0.9999	4.87×10^{-5}	2.8675	0.0674	0.9124
	373	1.0000	1.67×10^{-5}	2.8885	0.0289	0.8988
**5A-ODPA, 0.01 mol/L***	298	0.9942	5.74×10^{-3}	3.7049	0.4784	0.8269
	323	0.9989	8.34×10^{-4}	3.5014	0.2063	0.8316
	348	0.9999	9.42×10^{-5}	3.4716	0.0659	0.9056
	373	1.0000	1.54×10^{-5}	3.6914	0.0273	0.8869
**5A-ODPA, 0.005 mol/L**	298	0.9955	4.74×10^{-3}	3.9054	0.4838	0.7766
	323	0.9990	9.09×10^{-4}	3.6893	0.1970	0.8481
	348	1.0000	2.56×10^{-5}	3.6206	0.0635	0.9220
	373	1.0000	1.64×10^{-5}	3.9702	0.0271	0.8715
**5A-ODPA, 0.005 mol/L***	298	0.9966	4.08×10^{-3}	4.2752	0.5835	0.7375
	323	0.9977	2.27×10^{-3}	3.9358	0.2425	0.7874
	348	1.0000	4.85×10^{-5}	3.9297	0.0715	0.9174
	373	0.9999	3.81×10^{-5}	4.1433	0.0298	0.8926
**5A-ODPA, 0.001 mol/L**	298	0.9962	4.02×10^{-3}	3.9142	0.4844	0.7931
	323	0.9988	1.13×10^{-3}	3.8219	0.2016	0.8421
	348	0.9998	1.81×10^{-4}	3.6485	0.0692	0.9198
	373	1.0000	6.51×10^{-6}	3.8450	0.0275	0.8946
**5A-ODPA, 0.001 mol/L***	298	0.9989	1.54×10^{-3}	4.3728	0.6561	0.8328
	323	0.9991	1.09×10^{-3}	4.3784	0.2127	0.8547
	348	0.9999	7.42×10^{-5}	4.2891	0.0746	0.9061
	373	1.0000	2.31×10^{-5}	4.4438	0.0270	0.9141

Table S4 Adsorption isotherms parameters of the Sips model for C_3H_6 adsorption in pristine and modified 5A zeolite. (The asterisk (*) denotes THF-washed materials.)

Material	Temperature (K)	Adj. R^2	ΔQ	Model parameters		
				q_m (mmol/g)	k_S (kPa^{-1})	n
pristine zeolite 5A	298	0.9871	3.42×10^{-3}	2.0534	1.0959	0.7710
	323	0.9895	2.29×10^{-3}	1.8632	0.3794	0.8621
	348	0.9944	1.20×10^{-3}	1.9542	0.2304	0.7018
	373	0.9740	4.94×10^{-3}	1.6403	0.1371	1.0490
5A-ODPA, 0.01 mol/L	298	0.9932	4.57×10^{-4}	2.1721	0.0706	0.5492
	323	0.9849	7.34×10^{-4}	0.7888	0.0500	1.3143
	348	0.9870	4.03×10^{-4}	0.7765	0.0254	1.0447
	373	0.9845	3.59×10^{-4}	0.6620	0.0199	1.3158
5A-ODPA, 0.01 mol/L*	298	0.9902	9.94×10^{-4}	1.3660	0.1278	0.7892
	323	0.9934	5.72×10^{-4}	1.3037	0.1076	0.7300
	348	0.9881	8.66×10^{-4}	1.2488	0.0895	0.6649
	373	0.9964	2.15×10^{-4}	1.0819	0.0592	0.8034
5A-ODPA, 0.005 mol/L	298	0.9947	2.60×10^{-3}	1.1767	0.3311	0.7402
	323	0.9947	1.90×10^{-3}	1.1378	0.2437	0.7124
	348	0.9963	3.95×10^{-4}	0.9625	0.1066	0.6858
	373	0.9925	1.54×10^{-4}	0.9040	0.0392	0.7220
5A-ODPA, 0.005 mol/L*	298	0.9947	8.87×10^{-4}	1.2980	0.1226	1.4981
	323	0.9947	8.39×10^{-4}	1.2125	0.0912	1.3806
	348	0.9963	4.02×10^{-4}	1.1732	0.0591	0.9343
	373	0.9925	6.59×10^{-4}	0.9239	0.0430	1.2468
5A-ODPA, 0.001 mol/L	298	0.9936	1.18×10^{-3}	1.6996	0.2337	0.9220
	323	0.9935	6.62×10^{-4}	1.5600	0.1171	0.5827
	348	0.9804	1.27×10^{-3}	1.0559	0.0746	0.8578
	373	0.9938	6.18×10^{-4}	1.4459	0.0521	0.7725
5A-ODPA, 0.001 mol/L*	298	0.9932	2.21×10^{-3}	2.1361	0.3882	1.3164
	323	0.9933	9.71×10^{-4}	1.6307	0.2904	0.6911
	348	0.9920	9.49×10^{-4}	1.4590	0.1684	0.7829
	373	0.9976	3.74×10^{-4}	1.4350	0.0973	0.8511

Table S5 Adsorption isotherms parameters of the Langmuir model for CO_2 adsorption in pristine and modified 5A zeolite. (The asterisk (*) denotes THF-washed materials.)

Material	Temperature (K)	Adj. R^2	ΔQ	Model parameters	
				q_m (mmol/g)	k_L (kPa^{-1})
pristine zeolite 5A	298	0.9909	1.24×10^{-2}	4.1938	0.5982
	323	0.9979	2.73×10^{-3}	4.0918	0.1906
	348	0.9992	8.60×10^{-4}	4.0287	0.0638
	373	0.9984	1.22×10^{-3}	3.9881	0.0249
5A-ODPA, 0.01 mol/L	298	0.9939	3.91×10^{-3}	2.9975	0.2943
	323	0.9969	1.65×10^{-3}	2.8091	0.1581
	348	0.9995	2.51×10^{-4}	2.7324	0.0581
	373	0.9994	1.76×10^{-4}	2.5944	0.0248
5A-ODPA, 0.01 mol/L*	298	0.9916	8.22×10^{-3}	3.5778	0.4342
	323	0.9967	2.49×10^{-3}	3.3191	0.1660
	348	0.9993	3.63×10^{-4}	3.2887	0.0562
	373	0.9993	3.35×10^{-4}	3.2464	0.0231
5A-ODPA, 0.005 mol/L	298	0.9911	9.34×10^{-3}	3.7163	0.4275
	323	0.9972	2.50×10^{-3}	3.5132	0.1621
	348	0.9996	3.19×10^{-4}	3.4648	0.0556
	373	0.9992	4.29×10^{-4}	3.4009	0.0227
5A-ODPA, 0.005 mol/L*	298	0.9910	1.09×10^{-2}	4.0424	0.5294
	323	0.9943	5.53×10^{-3}	3.6697	0.1908
	348	0.9995	4.54×10^{-4}	3.7640	0.0621
	373	0.9994	4.00×10^{-4}	3.6956	0.0254
5A-ODPA, 0.001 mol/L	298	0.9926	7.94×10^{-3}	3.7422	0.4349

	323	0.9968	3.06 ×10^{-3}	3.6327	0.1653
	348	0.9993	5.29 ×10^{-4}	3.4990	0.0603
	373	0.9994	3.00 ×10^{-4}	3.4270	0.0234
**5A-ODPA, 0.001 mol/L***	298	0.9962	5.27 ×10^{-3}	4.2555	0.6237
	323	0.9974	2.25 ×10^{-3}	4.1936	0.1772
	348	0.9993	6.97 ×10^{-4}	4.0884	0.0635
	373	0.9996	2.95 ×10^{-4}	4.0484	0.0238

Table S6 Adsorption isotherms parameters of the Langmuir model for C_3H_6 adsorption in pristine and modified 5A zeolite. (The asterisk (*) denotes THF-washed materials.)

Material	Temperature (K)	Adj. R^2	ΔQ	Model parameters	
				q_m (mmol/g)	k_L (kPa^{-1})
**pristine zeolite 5A**	298	0.9826	4.60 ×10^{-3}	1.9891	0.4829
	323	0.9888	2.44 ×10^{-3}	1.8084	0.3873
	348	0.9870	2.80 ×10^{-3}	1.7843	0.2738
	373	0.9755	4.65 ×10^{-3}	1.6632	0.1331
**5A-ODPA, 0.01 mol/L**	298	0.97872	1.42 ×10^{-3}	1.1654	0.0456
	323	0.98233	8.60 ×10^{-4}	0.9061	0.0379
	348	0.98787	3.75 ×10^{-4}	0.8085	0.0232
	373	0.9828	3.99 ×10^{-4}	0.9508	0.0102
**5A-ODPA, 0.01 mol/L***	298	0.98857	1.16 ×10^{-3}	1.2706	0.1710
	323	0.98819	1.02 ×10^{-3}	1.1730	0.1475
	348	0.97811	1.59 ×10^{-3}	1.0909	0.1055
	373	0.99427	3.45 ×10^{-4}	0.9749	0.0753
**5A-ODPA, 0.005 mol/L**	298	0.96015	3.80 ×10^{-3}	1.1017	0.3755
	323	0.96471	2.44 ×10^{-3}	1.0449	0.2850
	348	0.98234	7.64 ×10^{-4}	0.8410	0.1432
	373	0.9904	3.11 ×10^{-4}	0.7453	0.0643
**5A-ODPA, 0.005 mol/L***	298	0.98925	2.30 ×10^{-3}	1.4311	0.1135
	323	0.99166	1.33 ×10^{-3}	1.3054	0.0991
	348	0.99647	3.90 ×10^{-4}	1.1418	0.0531
	373	0.99062	8.29 ×10^{-4}	1.0085	0.0266
**5A-ODPA, 0.001 mol/L**	298	0.99375	1.17 ×10^{-3}	1.6711	0.2372
	323	0.97869	2.18 ×10^{-3}	1.2895	0.1842
	348	0.97988	1.30 ×10^{-3}	0.9898	0.0865
	373	0.99056	9.42 ×10^{-4}	1.2624	0.0728
**5A-ODPA, 0.001 mol/L***	298	0.98957	3.21 ×10^{-3}	2.2004	0.4016
	323	0.98657	1.97 ×10^{-3}	1.5038	0.3126
	348	0.98834	1.39 ×10^{-4}	1.3642	0.1879
	373	0.99635	5.73 ×10^{-4}	1.3521	0.1105

Table S7. Thermodynamic parameters for C_3H_6 adsorption in pristine and the ODPA-modified zeolite 5A material. (The asterisk (*) denotes THF-washed materials.)

Material	ΔH^0 (kJ/mol)	ΔS^0 (J/mol·K)	ΔG^0 (kJ/mol)			
			298 K	323 K	348 K	373 K
**Pristine zeolite 5A**	−25.12	−46.10	−11.38	−10.22	−9.07	−7.92
**5A−ODPA, 0.01 mol/L***	−9.06	−8.60	−6.46	−6.25	−6.03	−5.81
**5A−ODPA, 0.01 mol/L**	−16.54	−38.90	−4.96	−3.98	−3.01	−2.04
**5A−ODPA, 0.005 mol/L***	−13.16	−22.90	−6.31	−5.74	−5.17	−4.59
**5A−ODPA, 0.005 mol/L**	−26.25	−57.10	−9.23	−7.80	−6.37	−4.94
**5A−ODPA, 0.001 mol/L***	−17.15	−26.20	−9.32	−8.66	−8.01	−7.35
**5A−ODPA, 0.001 mol/L**	−18.42	−35.90	−7.69	−6.79	−5.89	−4.99

Table S8 The diffusion parameters for CO_2 adsorption in pristine and the ODPA-modified zeolite 5A material at 298 K. The asterisks (*) indicate the THF-washed materials.

Material	External film diffusion			Internal diffusion			Adsorption equilibrium		
	k_{diff1}	C	Adj. R^2	K_{diff2}	C	Adj. R^2	K_{diff3}	C	Adj. R^2
**Pristine zeolite 5A**	18.21	-8.87	0.98943	2.27	1.76	0.902	6.47×10^{-15}	4.10	1.000
**5A-ODPA, 0.01 mol/L***	10.39	-5.22	0.98876	1.80	1.49	0.950	8.06×10^{-2}	3.36	0.800
**5A-ODPA, 0.01 mol/L**	6.41	-3.44	0.98632	1.04	1.41	0.904	1.48×10^{-13}	2.75	1.000
**5A-ODPA, 0.005 mol/L***	13.07	-6.38	0.99045	1.88	2.02	0.896	-8.49×10^{-14}	4.01	1.000
**5A-ODPA, 0.005 mol/L**	11.16	-5.86	0.98346	1.88	1.48	0.923	1.40×10^{-1}	3.43	0.909
**5A-ODPA, 0.001 mol/L***	14.31	-7.33	0.98807	1.99	2.04	0.908	7.23×10^{-14}	4.23	1.000
**5A-ODPA, 0.001 mol/L**	11.64	-6.15	0.98588	1.69	1.76	0.918	6.71×10^{-14}	3.71	1.000

Table S9 The diffusion parameters for C_3H_6 adsorption in pristine and the ODPA-modified zeolite 5A material at 298 K. The asterisks (*) indicate the THF-washed materials.

Material	External film diffusion			Internal diffusion			Adsorption equilibrium		
	k_{diff1}	C	Adj. R^2	k_{diff1}	C	Adj. R^2	k_{diff1}	C	Adj. R^2
**Pristine zeolite 5A**	9.64	-5.20	0.966	0.30	1.53	0.907	3.69×10^{-16}	2.05	1.000
**5A-ODPA, 0.01 mol/L***	1.38	-0.85	0.993	0.46	0.54	0.940	8.49×10^{-16}	1.47	1.000
**5A-ODPA, 0.01 mol/L**	0.24	-0.12	0.956	0.70	-0.63	0.992	8.51×10^{-16}	1.00	1.000
**5A-ODPA, 0.005 mol/L***	1.51	-0.48	0.990	0.28	0.79	0.962	7.02×10^{-17}	1.33	1.000
**5A-ODPA, 0.005 mol/L**	1.07	-0.78	0.981	0.43	0.26	0.973	-3.57×10^{-16}	1.20	1.000
**5A-ODPA, 0.001 mol/L***	2.17	-1.14	0.997	0.50	0.97	0.937	-1.26×10^{-16}	1.98	1.000
**5A-ODPA, 0.001 mol/L**	2.65	-1.51	0.994	0.36	1.21	0.911	1.47×10^{-17}	1.87	1.000
